# How Microbiome Composition Correlates with Biochemical Changes during Sauerkraut Fermentation: a Focus on Neglected Bacterial Players and Functionalities

**DOI:** 10.1128/spectrum.00168-22

**Published:** 2022-06-14

**Authors:** Ali Zein Alabiden Tlais, Wilson José Fernandes Lemos Junior, Pasquale Filannino, Stefano Campanaro, Marco Gobbetti, Raffaella Di Cagno

**Affiliations:** a Faculty of Science and Technology, Libera Università di Bolzano, Bolzano, Italy; b Department of Soil, Plant and Food Sciences, University of Bari A. Moro, Bari, Italy; c Department of Biology, University of Padova, Padova, Italy; Universitá of Roma Tre

**Keywords:** microbiota, sauerkraut fermentation, *Secundilactobacillus malefermentans*, phenolics profile

## Abstract

This study provided a new perspective on the bacterial community succession during sauerkraut fermentation, and on resulting metabolic functions. While culture-dependent methods confirmed the key role of the well-known core microbiome species, metagenomic approach (shotgun) revealed *Secundilactobacillus malefermentans* as a species of the core microbiome, especially during the last weeks of fermentation. Although the potentiality of *S. malefermentans* has not yet fully explored, it held core functional genes usually attributed to others lactic acid bacteria driving sauerkraut fermentation. Based on our results it is arguable that *S. malefermentans* might have a key a role during sauerkraut fermentation carried out at low temperature. Under our experimental conditions, the profile of phenolic compounds changed throughout sauerkraut fermentation. The amount of free phenolics, including free phenolic acids, increased at the beginning of the fermentation, whereas the conversion of phenolic acids into microbial derivatives was consistent during the last part of the sauerkraut fermentation. We pioneered correlating changes in the phenolics profile to changes in the microbiome, although the framework presented is still fragmentary. Annotated genes linked to the phenolic compounds metabolism (*VprA* and *padA*) were found in many core species during the whole process. A high metabolic potential for phenolics bioconversion emerged for lactobacilli and *Pediococcus* spp. through correlation analysis between microbiome composition and phenolics profile.

**IMPORTANCE** Our study was not limited to describe the succession pattern of the microbial community during sauerkraut fermentation, but also revealed how some neglected bacterial players belong to the core species during sauerkrauts processing, especially at low temperature. Such species might have a role as potential starters to optimize the fermentation processes and to obtain sauerkrauts with improved and standardized nutritional and sensory features. Furthermore, our correlations between microbiome composition and phenolics profile might also represent new references for sauerkraut biotechnology, aiming to identify new metabolic drivers of potential sauerkraut functionalities. Finally, sauerkraut ecosystem is a tractable model, although with high level of complexity, and resultant ecological information might be extended to other plant ecosystems.

## INTRODUCTION

Plant-associated microbial communities do not represent random assemblages but reveal defined phylogenetic organization (1). When plant-based matrices undergo fermentation, the resulting microbial assembly is a successional, multistep process governed by microorganisms dispersal and interactions, and plant-related and environmental drivers, with pH reduction and acid resistance being prevalent (2). Developing robust models of microbiome structure to address what ecological factors affect its assembly and functionality represents a current scientific challenge in the field of food fermentations. Lactic acid bacteria are the key microbial players during sauerkraut fermentation. Despite their low abundances in raw cabbage and the variability associated with the farming regions, spontaneous fermentation proceeds in a well-defined way (3). This is due to the pressure of stochastic, temporal and, most importantly, deterministic drivers that shape the structure of the microbiota in relation to the functionality of the microbiome.

Given that sauerkraut is a result of spontaneous lactic acid fermentation of cabbages, its nutritional and sensory features are strictly dependent on the resident microbial community and successful fermentation conditions (4–6). Most of the mechanistic knowledge with respect to the identity and functionality of microbes that harbor spontaneous fermented sauerkraut has gained through culturing, even though several bacteria are recalcitrant to isolation using common culturing methods, which leads to an underestimation of the subdominant population. Studies on succession of the microbiota reported *Leuconostoc* sp. and lactobacilli as the dominant microorganisms in the early and middle/late stages of sauerkraut fermentation, respectively (7, 8). Previous ecological studies indicated temperature and salt concentration as the main effectors driving the biodiversity of sauerkraut and other fermented vegetables (7, 9–11). Besides, other studies linked sauerkraut microbial succession to the acidification occurring especially during the early phase of fermentation (8, 9, 12). Sauerkraut metabolite composition reflects the global phenotype of the whole microbial community (13). The interconnections between volatile metabolites, organic acids, and microbial diversity in sauerkraut have already been described highlighting the main microbial genera contributing to flavor (8). Conversely, the causal effect of secondary plant metabolites on microbial communities in sauerkraut has been neglected. For instance, bacterial growth and viability are diversely affected in various fermented vegetables depending on the chemical structure and concentration of phenolic compounds (14). Some lactic acid bacteria (LAB) showed the capability to tolerate high levels of phenolic compounds, such as *Lactiplantibacillus plantarum*, *Weissella* spp., Levilactobacillus brevis, and Leuconostoc mesenteroides, and this ability was correlated with the chance of such species to metabolize these compounds (15). The metabolism of phenolic compounds by LAB was proposed as a detoxification mechanism (16) or as a tool to maintain the NAD/NADH balance (17, 18) and to provide additional metabolic energy through a chemiosmotic mechanism (19). On the other side, microbial derivatives from phenolics metabolism may have significant impacts on the sensory and health-promoting features of fermented vegetables (20). In this study, we aimed at establishing not yet disclosed correlations between LAB microbiome composition and phenolic compounds occurring throughout sauerkraut spontaneous fermentation, which might be identified as new metabolic drivers of potential sauerkraut functionalities. Through sensitive approaches based on mass spectrometry-based metabolomics and next generation sequencing-based metagenomics, we also aim to shift the spotlights on LAB species whose role was so far neglected during sauerkraut fermentation, also evaluating the presence of annotated genes correlated with phenolics metabolic pathway. Once shaped, the diversity of the sauerkraut microbiota functionality might reflect on sauerkraut biotechnology.

## RESULTS

### Physicochemical and microbiological changes throughout sauerkraut fermentation.

Samples were taken at the beginning of fermentation (D0), after 0.5 (D0.5), 1 (D1), 2 (D2), 3 (D3), 4 (D4), 5 (D5), 7 (D7), 14 (D14), 21 (D21), 28 (D28), 35 (D35), and 42 (D42) days of fermentation. Raw white cabbage had initial value of pH of ca. 5.87 ± 0.01, which progressively decreased during the first week of fermentation (3.93 ± 0.02) and a further slight decrease was found at the end of the fermentation (3.36 ± 0.02) (Table 1). Accordingly, total titratable acidity (TTA) significantly increased (*P* < 0.05) (Table 1).

**TABLE 1:** Values of pH, total titratable acidity (TTA) (mL 0.1 M NaOH 10 g^−1^ DW), and concentration (mg g^−1^ DW) of glucose, fructose, mannitol, lactic, acetic, and citric acids during spontaneous fermentation of sauerkraut carried out at 15°C for 42 days^*a*^

Time (days)	D0^*b*^	D0.5	D1	D2	D3	D4	D5	D7	D14	D21	D28	D35	D42
pH	5.87 ± 0.01^a^^,^^b^	5.76 ± 0.02^b^	5.71 ± 0.03^b^	5.70 ± 0.11^b^	5.15 ± 0.07^c^	4.69 ± 0.1^d^	4.36 ± 0.05^e^	3.93 ± 0.02^f^	3.74 ± 0.06^f^^,^^g^	3.67 ± 0.02^g^^,^^h^	3.54 ± 0.01^g,h,i^	3.49 ± 0.01^h,i^	3.36 ± 0.02^i^
TTA (mL 0.1 M NaOH 10 g^−1^ DW)	0.91 ± 0.02^i^	0.95 ± 0.07^i^	1.05 ± 0.07^i^	1.30 ± 0.00^h^^,^^i^	2.25 ± 0.21^h^	3.55 ± 0.07^g^	4.20 ± 0.14^g^	5.90 ± 0.14^f^	8.75 ± 0.49^e^	10.9 ± 0.57^d^	13.8 ± 0.00^c^	15.05 ± 0.07^b^	16.9 ± 0.42^a^
Glucose (mg g^−1^ DW)	217.9 ± 0.33^a^	215.4 ± 0.85^b^	204.9 ± 1.17^c^	203.2 ± 0.15^c^^,^^d^	173.6 ± 0.64^e^	202.0 ± 0.25^d^	174.4 ± 0.55^e^	163.2 ± 0.57^f^	163.5 ± 0.6^f^	149.2 ± 0.38^g^	118.7 ± 0.27^j^	121.9 ± 0.60^i^	124.4 ± 0.2^h^
Fructose (mg g^−1^ DW)	104.5 ± 0.70^a^	98.4 ± 0.40^b^	95.4 ± 0.23^c^	89.8 ± 0.18^d^	74.9 ± 0.38^e^	86.1 ± 0.30^f^	64.8 ± 0.25^g^	34.3 ± 0.31^i^	32.4 ± 0.36^j^	55.6 ± 0.29^h^	22.9 ± 0.13^k^	18.9 ± 0.63^l^	11.1 ± 0.12^m^
Mannitol (mg g^−1^ DW)	0 ± 0.0^h^	0 ± 0.0^h^	0 ± 0.0^h^	0 ± 0.0^h^	5.7 ± 0.51^g^	23.4 ± 0.56^f^	36.2 ± 0.23^e^	79.3 ± 1.00^b^	86.2 ± 0.58^a^	34.1 ± 0.29^e^	53.5 ± 0.42^d^	21.1 ± 0.91^f^	64.0 ± 1.26^c^
Lactic acid (mg g^−1^ DW)	0.36 ± 0.08^k^	0.06 ± 0.08^k^	1.9 ± 0.14^j^	2.3 ± 0.03^j^	4.8 ± 0.3^i^	17.1 ± 0.21^h^	24.1 ± 0.36^g^	82.4 ± 0.28^f^	118.5 ± 0.66^e^	136.3 ± 0.36^c^	129.5 ± 0.37^d^	182.5 ± 0.52^b^	199.4 ± 0.21^a^
Acetic acid (mg g^−1^ DW)	1.50 ± 0.05^g^	2.58 ± 0.01^f^	3.72 ± 0.07^e^	4.12 ± 0.04^d^	3.73 ± 0.19^e^	5.01 ± 0.03^b^	4.74 ± 0.02^b^^,^^c^	4.90 ± 0.01^b^^,^^c^	7.12 ± 0.16^a^	4.54 ± 0.16^c^	5.04 ± 0.08^b^	3.77 ± 0.04^e^^,^^d^	4.76 ± 0.06^b^^,^^c^
Citric acid (mg g^−1^ DW)	7.60 ± 0.13^c^	8.62 ± 0.09^b^	2.65 ± 0.31^e^	3.06 ± 0.08^e^	5.98 ± 0.03^d^	9.76 ± 0.07^a^	7.76 ± 0.16^c^	0.09 ± 0.01^g^	0.07 ± 0.01^g^	0.28 ± 0.05^f^^g^	0.60 ± 0.06^f^	0.42 ± 0.02^f^^,^^g^	0.05 ± 0.00^g^

a Samples were taken at the beginning of fermentation (D0), after D0.5, D1, D2, D3, D4, D5, D7, D14, D21, D28, D35, and D42 of fermentation. Tukey’s test was used to determine significant differences among means at an error probability of 5% (*P* < 0.05).

b Means within the columns with different superscript letters (^a-m^) are significantly different (*P* < 0.05). The data are the means of three independent experiment ± standard deviations (*n* = 3).

Glucose and fructose, the predominant carbohydrates of raw white cabbage (217.9 ± 0.33 and 104.5 ± 0.3 mg 100 g^−1 ^dry weight [DW], respectively), significantly decreased (*P* < 0.05) over time, but they were still detectable after D42 fermentation (ca. 50 and 10%, respectively) (Table 1). Concomitantly with fructose consumption, mannitol was released from the third day of fermentation onwards. Main microbial metabolites were lactic and acetic acids (Table 1). During the first 3 days, a slight but significant increase of lactic acid was found, which markedly increased at each further time point until the end of the fermentation. Compared with the lactic acid, acetic acid gradually increased during the first 14 days of fermentation (7.1 ± 0.16 mg 100 g^−1^ DW) then decreased after 1 week (4.5 ± 0.16 mg 100 g^−1^ DW) and remained almost constant throughout the time. Citric acid was almost completely metabolized after 7 days of fermentation.

### Evolution of total and free phenolic compounds.

Total phenolic compounds were determined using methanol/water-soluble extract (MWSE). Raw white cabbage had a concentration of 311.0 ± 38 mg gallic acid eq. 100 g^−1^ DW. This value began to increase significantly (*P* < 0.05) after 4 days of fermentation and reached the highest concentration at the end of the fermentation (475.0 ± 5 mg gallic acid eq. 100 g^−1^ DW). Aiming to evaluate the effects of spontaneous fermentation on free phenolic compounds, their profile was analyzed through LC-MS/MS and HPLC-PAD analysis (Table 2). The highest peaks were identifiable using external standards. Sinapic acid was the most abundant phenolic acid (102.6 ± 1.16 μg g^−1^ DW) of raw cabbage. The spontaneous fermentation resulted in a marked reduction of sinapic acid during the first 3 days of fermentation (7.2 ± 1.58 μg g^−1^ DW). However, a gradual increase was observed from 3 to 5 days (85.0 ± 3.07 μg g^−1^ DW), then it decreased again up to 26.9 ± 2.09 μg g^−1^ DW (D42). The amount of ferulic, *p*-coumaric, and caffeic acids markedly increased after 1 day of fermentation, and then fluctuating concentrations were found up to 5 days. After that, all these compounds progressively decreased, with *p*-coumaric acid resulting completely metabolized. The highest levels of chlorogenic and (E)-cinnamic acids were found at D7 (10.1 ± 0.1 μg g^−1^ DW) and D1 (19.21 ± 1.39 μg g^−1^ DW), respectively, and then gradually decreased throughout the fermentation. Vanillin did not significantly change (*P* > 0.05) compared with raw cabbage. Kaempferol was found at the highest concentration after 1 week of fermentation (4.8 ± 0.10 μg g^−1^ DW) but slowly decreased in the following 2 weeks, and then remained almost constant until the end of the fermentation (3.9 ± 0.06 μg g^−1^ DW). Spontaneous fermentation affected the levels of microbial derivatives of phenolic acids during time (Table 2). Dihydrocaffeic and 4-ethyl catechol were found at the highest amount at D2 (7.9 ± 0.18 μg g^−1^ DW) and D7 (7.1 ± 0.04 μg g^−1^ DW), respectively, and then slowly decreased throughout the fermentation. Overall, the concentration of phloretic acid gradually increased from 3.2 ± 0.04 μg g^−1^ DW (D0) to 6.3 ± 0.37 μg g^−1^ DW (D42). Dihydroferulic acid reached the highest level after 21 days of fermentation, and then remained almost constant until the end of the fermentation (Table 2).

**TABLE 2:** Quantification of phenolic compounds (μg g^−1^ DM) by LC-MS/MS and HPLC-PAD in methanol-water soluble extract (MWSE) from sauerkraut fermented at 15°C for 42 days^*a*^

Time (days)	D0^*b*^	D0.5	D1	D2	D3	D4	D5	D7	D14	D21	D28	D35	D42
Chlorogenic acid	5.87 ± 0.19^g^	7.33 ± 0.26^c^^,^^d^^,^^e^	5.15 ± 0.01^h^	3.92 ± 0.03^i^	3.48 ± 0.10^i^	6.59 ± 0.12^f^	7.48 ± 0.06^c^^,^^d^	10.10 ± 0.10^a^	8.72 ± 0.07^b^	6.99 ± 0.10^e^^,^^f^^,^^g^	7.65 ± 0.07^c^	6.84 ± 0.15^e^^,^^f^	7.08 ± 0.04^d^^,^^e^^,^^f^
Caffeic acid	0.55 ± 0.09^b^	0.01 ± 0.002^c^	2.34 ± 0.21^a^	0.04 ± 0.02^d^^c^	n.d.^*c*^	0.30 ± 0.01^b^^,^^c^^,^^d^	0.58 ± 0.08^b^	n.d.	0.02 ± 0.00^e^	0.01 ± 0.00^e^	0.01 ± 0.00^e^	0.06 ± 0.02^c^^,^^d^^,^^e^	0.34 ± 0.09^b^^,^^c^
*p*-coumaric acid	1.97 ± 0.41^a^^,^^b^	0.41 ± 0.45^b^^,^^c^	3.51 ± 0.44^a^	0.59 ± 0.08^b^^,^^c^	0.13 ± 0.02^b^^,^^c^	0.85 ± 0.34^b^^,^^c^	0.97 ± 0.21^b^^,^^c^	n.d.	n.d.	n.d.	n.d.	n.d.	n.d.
Vanillin	3.38 ± 0.43^a^^,^^b^	4.19 ± 0.40^a^	3.72 ± 0.17^a^^,^^b^	3.72 ± 0.62^a^^,^^b^	3.67 ± 0.3^a^^,^^b^	2.85 ± 0.31^b^	3.37 ± 0.10^a^^,^^b^	3.02 ± 0.38^a^^,^^b^	3.08 ± 0.12^a^^,^^b^	2.80 ± 0.13^b^	3.05 ± 0.26^a^^,^^b^	3.30 ± 0.23^a^^,^^b^	3.65 ± 0.03^a^^,^^b^
Ferulic acid	7.95 ± 0.06^c^	5.96 ± 0.02^d^	13.03 ± 0.71^a^	4.89 ± 0.03^d^^,^^e^	4.81 ± 0.32^d^^,^^e^	8.81 ± 0.56^b^^,^^c^	9.93 ± 0.37^b^	5.23 ± 0.06^d^^,^^e^	4.74 ± 0.00^e^	4.64 ± 0.22^e^	4.77 ± 0.24^d^^,^^e^	4.91 ± 0.09^d^^,^^e^	4.94 ± 0.09^d^^,^^e^
Sinapic acid	102.62 ± 3.16^a^	54.73 ± 1.92^c^^,^^d^	71.51 ± 0.72^b^^,^^c^	12.47 ± 0.57^f^^,^^g^	7.20 ± 1.58^g^	72.42 ± 14.81,^b^^c^	84.98 ± 3.07^a^^,^^b^	55.31 ± 6.80^c^^,^^d^	18.84 ± 2.34^f^^,^^g^	25.18 ± 1.77^e^^,^^f^^,^^g^	69.62 ± 0.72^b^^,^^c^	42.55 ± 0.62^d^^,^^e^	26.92 ± 2.09^e^^,^^f^
(E)-cinnamic acid	2.26 ± 0.23^d^	7.88 ± 2.31^b^^,^^c^	19.21 ± 1.39^a^	16.40 ± 2.07^a^	9.36 ± 2.33^b^	4.17 ± 1.71^b^^,^^c^^,^^d^	3.33 ± 0.54^c^^,^^d^	1.68 ± 0.03^d^	1.62 ± 0.38^d^	2.36 ± 1.43^d^	1.29 ± 0.27^d^	1.47 ± 0.38^d^	1.15 ± 0.07^d^
Kaempferol	4.02 ± 0.17^b^^,^^c^	3.41 ± 0.02^c^	3.86 ± 0.12^c^^,^^d^	3.44 ± 0.07^e^	3.50 ± 0.02^e^^,^^f^	3.63 ± 0.01^d^^,^^e^^,^^f^	3.83 ± 0.07^c^^,^^d^^,^^e^	4.78 ± 0.10^a^	4.29 ± 0.05^b^	3.82 ± 0.07^c^^,^^d^^,^^e^	3.84 ± 0.10^c^^,^^d^	3.84 ± 0.04^c^^,^^d^	3.90 ± 0.06^c^^,^^d^
Phloretic acid	3.24 ± 0.04^h^	3.54 ± 0.11^g^	3.79 ± 0.17^e^^,^^f^	3.84 ± 0.64^e^	3.63 ± 0.01^f^^,^^g^	2.76 ± 0.12^i^	3.31 ± 0.24^h^	5.22 ± 0.03^c^	5.49 ± 0.06^b^	4.98 ± 0.57^d^	5.88 ± 0.17^b^	5.24 ± 1.26^c^	6.30 ± 0.37^a^
Dihydrocaffeic acid	4.81 ± 0.02^f^^,^^g^	7.03 ± 0.02^b^	4.68 ± 0.06^f^^,^^g^	7.91 ± 0.18^a^	6.58 ± 0.04^c^	5.69 ± 0.04^d^	5.33 ± 0.06^e^	4.59 ± 0.09^g^	4.92 ± 0.01^f^	4.90 ± 0.07^f^	4.80 ± 0.01^f^^,^^g^	4.85 ± 0.06^f^^,^^g^	4.91 ± 0.08^f^
Dihydroferulic acid	2.24 ± 0.04^i^	5.83 ± 0.11^c^	2.34 ± 0.02^i^	3.73 ± 0.08^f^	3.44 ± 0.01^g^	3.08 ± 0.01^h^	3.19 ± 0.05^h^	4.99 ± 0.15^e^	5.39 ± 0.01^d^	6.91 ± 0.03^a^	6.38 ± 0.04^b^	6.56 ± 0.06^b^	6.84 ± 0.02^a^
4-ethyl catechol	4.36 ± 0.02^d^	5.85 ± 0.01^b^	3.29 ± 0.02^e^^,^^f^^,^^g^	5.09 ± 0.00^c^	2.89 ± 0.03^f^^,^^g^	1.77 ± 0.00^h^	1.77 ± 0.01^h^	7.10 ± 0.04^a^	5.16 ± 0.02^b^^,^^c^	2.73 ± 0.63^g^	3.45 ± 0.03^e^^,^^f^	3.25 ± 0.01^e^^,^^f^^,^^g^	3.64 ± 0.03^e^

a Samples were taken at the beginning of fermentation (D0), after D0.5, D1, D2, D3, D4, D5, D7, D14, D21, D28, D35, and D42 days of fermentation.

b Means within the columns with different superscript letters are significantly different (*P* < 0.05). The data are the means of three independent experiment ± standard deviations (*n* = 3).

c n.d., not detected.

### Sauerkraut microbiota as estimated by culturing.

As estimated on plate count agar (PCA) and malt extracts agar (MEA) media, the total bacterial and yeasts in raw cabbages were 5.7 ± 0.01 and 3.4 ± 0.12 Log CFU g^−1^, respectively. Although no LAB were found by culture-dependent method in 10 g of raw cabbages, the cell density of presumptive LAB sharply increased during the first 4 days of fermentation (8.4 ± 0.4 Log CFU g^−1^), and then remained almost constant until the end of the fermentation. The count of yeasts slightly increased (ca. a half log cycle) after D1, then progressively decreased until they disappeared at D35 of fermentation. The number of total bacteria almost mirrored that of LAB reaching the highest cell density at D5 (8.7 ± 0.06 Log CFU g^−1^), followed by a slight reduction throughout the fermentation.

**FIG 1:**
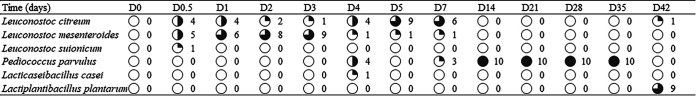
Species of lactic acid bacteria identified by partial sequencing of 16S rRNA during spontaneous fermentation of sauerkraut carried out at 15°C for 42 days. Samples were taken at the beginning of fermentation (D0), after D0.5, D1, D2, D3, D4, D5, D7, D14, D21, D28, D35, and D42 days of fermentation. Circles represent the percentage of isolates for each time point = 0% (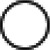); 1–30% (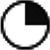); 31–59% (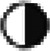); 60–90% (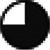); and 91–100% (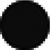).

Gram-positive, catalase-negative, nonmotile cocci and rods, able to acidify MRS broth, randomly isolated from the highest plate dilutions of each time point, were identified by partial sequencing using primer LpigF/LpigR (5′-TACGGGAGGCAGCAGTAG-3′ and 5′-CATGGTGTGACGGGCGGT-3′ as according to reference 21) corresponding to positions 369 to 386 and 1,424 to 1,441, respectively, of the 16S rRNA gene sequence. All 120 isolates underwent to RAPD-PCR analysis. At the linkage distance of 3.4, isolates gathered into 23 clusters (data not shown). Joining results from all time points of sauerkraut, six species of LABs were identifiable. The highest relative abundance was for Leuconostoc citreum (25.8% isolates), *Leuc. mesenteroides* (25.8%), Leuconostoc suionicum (0.8%), *P. parvulus* (39.2%), *Lacticaseibacillus casei* (0.8%), and *L. plantarum* (7.5%) whose succession over time was shown in Fig. 1. At D0, no LAB species were identified. During the first 3 days of fermentation, *Leuc. citreum* and *Leuc. mesenteroides* resulted as the dominant species. Subsequently (up to 7 days), *L. casei* and *P. parvulus* appeared, while in the interval of time between 14 and 35 days, *Leuc. citreum* and *Leuc. mesenteroides* disappeared and the environment was completely dominated by *P. parvulus.* At the end of fermentation, sauerkraut was mainly dominated by *L. plantarum* (Fig. 1).

### Sauerkraut microbiome as estimated by shotgun bacterial metagenome sequencing.

Using shotgun metagenome sequencing, we obtained a data set of 2,419 different contigs. Multilevel PCA (PC1 45% and PC2 28%) and PCoA (Axis 1 55% and Axis 2 30%) differentiated sauerkraut samples in three clusters according to the bacterial diversity. Samples fermented until 2 days were clustered together; those fermented from 3 to 7 days were grouped in a second cluster and, from 14 to 42 days in the third cluster (Fig. S1). PERMANOVA confirmed the diversity among the three clusters (*P* < 0.001). Analyzing altogether the shotgun metagenome sequencing results for sauerkraut samples at all time points, the entire bacterial diversity was explained according to the above clustering.

**FIG 2:**
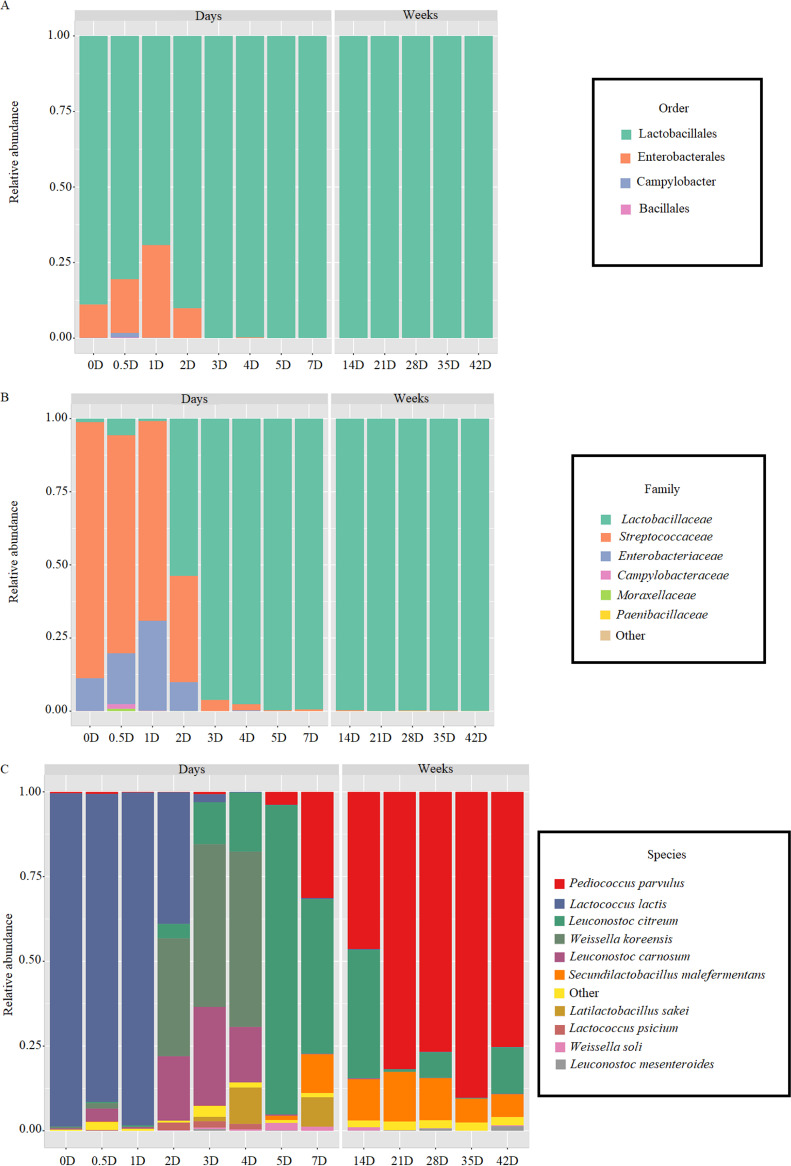
Relative abundance of bacterial order (A), family (B), and genus (C) level during spontaneous fermentation of sauerkraut carried out at 15°C for 42 days. Samples were taken at the beginning of fermentation (D0), after D0.5, D1, D2, D3, D4, D5, D7, D14, D21, D28, D35, and D42 of fermentation.

The succession of the microbiota at the order level during the spontaneous fermentation showed the raw cabbage (D0) the environment at highest bacterial diversity, with Lactobacillales (80.7%) and Enterobacterales (16.6%) as the predominant orders, while Campylobacterales (1.4%), Bacillales (0.26%), and Pseudomonadales (0.8%) were present at low relative abundance (Fig. 2A). During the first 3 days of fermentation, only Enterobacterales order was growing before it was replaced completely with Lactobacillales (99.9%) throughout the process (Fig. 2A). The order diversity reflected on the family and genus succession (Fig. 2B, C). In fact, 13 families were identified during the first 4 days of fermentation with *Streptococcaceae* (68.1%) as the major family followed by *Enteobacteriaceae* (30.7%). Then a dramatic change in the microbiota characterized the environment, which was mainly dominated by *Lactobacillaceae* (Fig. 2B). At the genus level, the highest diversity was found during the first 7 days with 22 genera, and then *P. parvulus* (46.5 up to 89.8%), *Leuconostoc* (0.14 up to 37.7%) and *S. malefermentans* (7.09 up to 12.08%) dominated the entire microbiota (Fig. 2C).

To further investigate the bacterial succession during spontaneous fermentation, taxonomic structures of bacterial communities at the species levels revealed 91 species of LAB (Fig. S1). Some of them coincided with those identified through culturing, but the diversity increased especially within *Companilactobacillus* sp., *Fructilactobacillus* sp., *Fructobacillus* sp., *Lactiplantibacillus* sp., *Lactobacillus* sp., *Latilactobacillus* sp., *Lentilactobacillus* sp., *Levilactobacillus* sp., *Ligilactobacillus* sp., *Limosilactobacillus* sp., *Loigolactobacillus* sp., *Paucilactobacillus* sp., and *Secundilactobacillus* sp. (Fig. S2). Twenty-eight species were identified in raw cabbages mainly distributed among *Leuconostoc* (five species), *Weissella* (five), *Lactobacillus* (three), *Pediococcus* (three), *Lactococcus* (two), *Levilactobacillus* (one), *Lactobacillus* sp. (one), *Latilactobacillus* sp., (one), and *Secundilactobacillus* sp. (one), together with some spoilage bacteria (nine). After 4 days of fermentation, the bacterial species diversity increased especially for lactobacilli (13 species), *Leuconostoc* (10), and *Weissella* (9). As the fermentation progressed, the number of species gradually decreased to reach 43 species at D42, where Pediococcus parvulus (65.7%), *Leuc. citreum* (13.5%) and *S. malefermentans* (7.1%) were the most abundant (Fig. S2).

**FIG 3:**
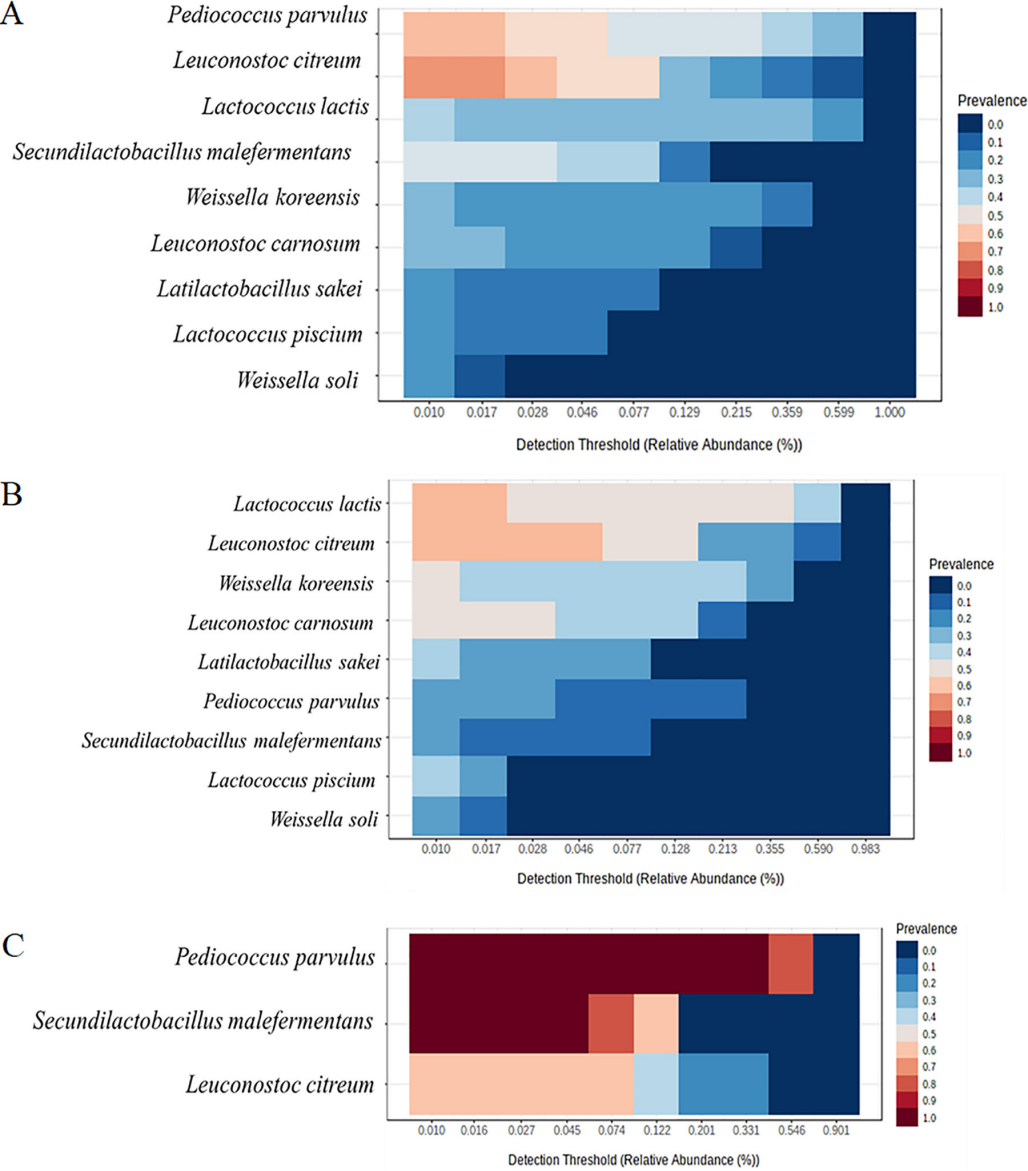
Core microbiome and relative abundances across lactic acid bacteria species during spontaneous fermentation of sauerkraut carried out at 15°C for 42 days. Samples were taken at the beginning of fermentation (D0), after D0.5, D1, D2, D3, D4, D5, D7, D14, D21, D28, D35, and D42 of fermentation. The core microbiome was calculated throughout the overall fermentation time (A), from D0 to D7 (B), and from D14 to D42 (C). The determination of core microbiota was carried out across evaluation abundance/prevalence thresholds with the blanket analysis (76) based on various signal and prevalence. The detection threshold of abundances set at 0.01% and prevalence above 20%. Red tonality shows the prevalence from 50% to 100% and blue tonality from 0% to 40%.

For the core microbiome analysis, the prevalence threshold was set to 20% and detection threshold of relative abundances was 0.01% (Fig. 3). The core microbiome comprised two species with 60% to 70% of prevalence (*Leuc. citreum* and *P. parvulus*) and seven with 50% or less prevalence, including *S. malefermentans*, Lactococcus lactis, and Weissella soli (Fig. 3A). Aiming to describe how the core microbiome changes during time, we considered two main intervals of time from D0 to D7 (Fig. 3B) and from D14 to D42 (Fig. 3C). As expected, during the first interval of time *Lc. lactis* and *Leuc. citreum* were found with 60% of prevalence followed by seven species with less than 50% of prevalence (Fig. 3B), whereas in the second period *S. malefermentans* and *P. parvulus* had 100% of prevalence followed by *Leuc. citreum* with 60% of prevalence (Fig. 3C).

### Microbiome functionality.

**FIG 4:**
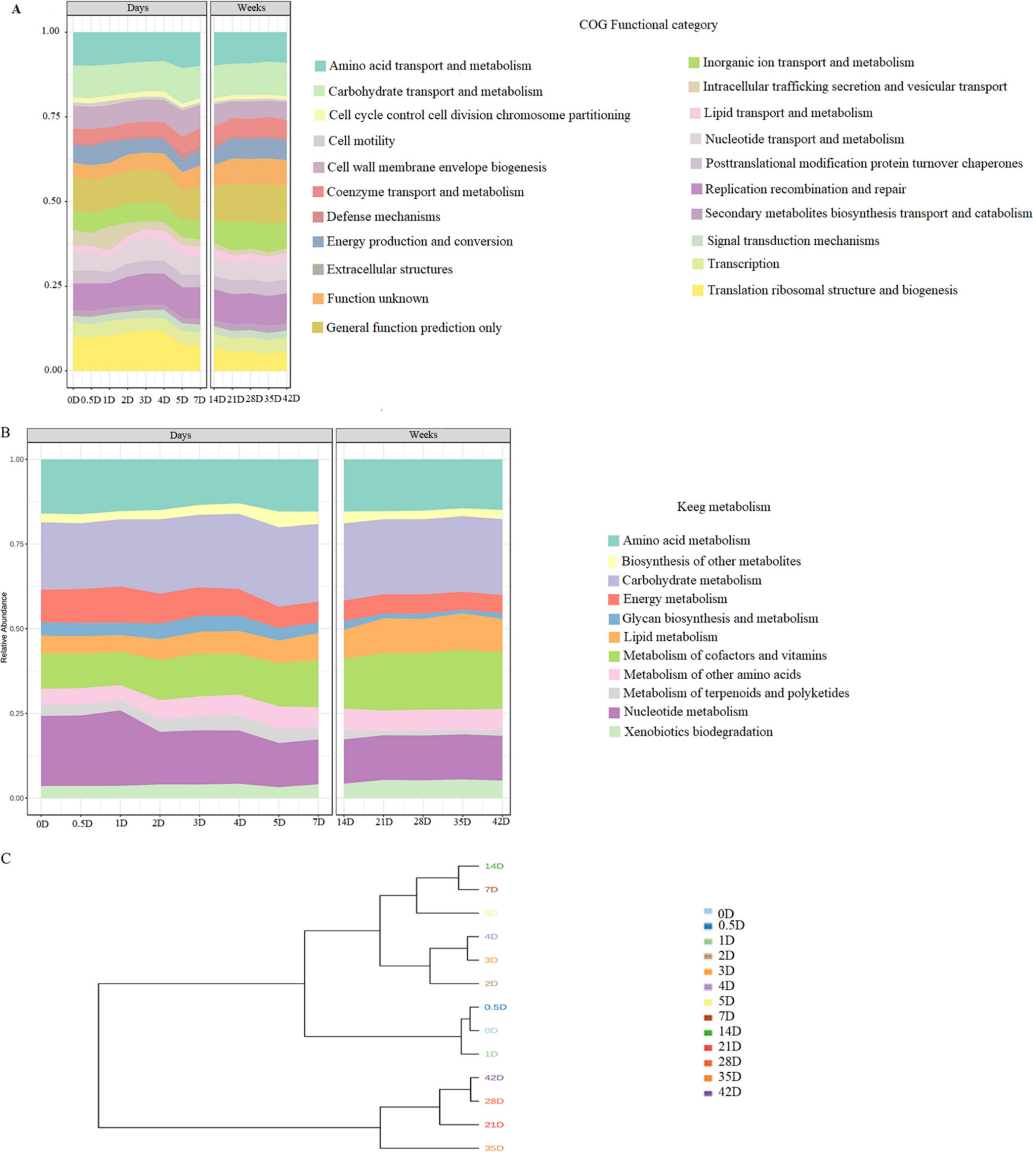
Profile of bacterial gene function (A), KEGG metabolism pathway (B), and dendrogram based on coverage frequency of annotated genes (C), during spontaneous fermentation of sauerkraut carried out at 15°C for 42 days. Samples were taken at the beginning of fermentation (D0), D0.5, D1, D2, D3, D4, D5, D7, D14, D21, D28, D35, and D42 of fermentation.

Shotgun metagenome analyses of the sauerkraut samples detected 66,429 genes annotated. The functions of bacterial genes and KEGG metabolism predicted by Prodigal revealed as mainly classified those related to carbohydrates, amino acids, nucleotides, and lipids metabolism and transport, extracellular structures, cells division and motility, transcription, translation, and other functions with lower abundance (Fig. 4A). At the beginning of the fermentation the nucleotides metabolism followed by carbohydrate, amino acids, cofactors and vitamins, and amino acids metabolisms were the most representative among the KEGG pathways. As fermentation proceeded, the genes related to nucleotide metabolism gradually decreased up to D5, then remained almost stable throughout the time. On the other hand, genes related to the metabolism of cofactors and vitamins and metabolism of lipids abounded in the last stages of fermentation (Fig. 4B).

The diversity among the KO revealed D3 and D4 as the time points with the highest number of unique KO, 29 and 28, respectively (data not shown). Unique enzyme related to the amino acid metabolism (K0003, Cysteine and methionine metabolism) was identified at D4, whose time point was the one with the highest gene functions. Metagenomics assembly showed 1,141 common enzyme among all time points reflecting the microbiome functionality of sauerkraut during the spontaneous fermentation.

Focusing on gene abundance throughout time, we determined which species showed the annotated gene core related to carbohydrate metabolism. The role of *Lc. lactis* (253 annotated genes), *Leuc. carnosum* (194), and *Leuc. citreum* (163) at the beginning of the fermentation (D0) and up to D4 for this metabolism was confirmed (Table S1). The functionality of *Leuc. citreum* for carbohydrates metabolism persisted during time. From the D4 onwards, the core gene annotated of *Leuc. citreum* was flanked by *S. malefermentans*, *Leuc. citreum*, and *P. parvulus*. The contribution of *Latilactobacillus sakei* was only limited to the interval between D3 and D7 of fermentation. These findings agreed the output of the nine metagenome assembled genomes (MAGs) obtained from binning, where all these species were identified with a completeness higher than 66% (Table S2). The contribution from each species to lipid metabolism was similar to that observed for carbohydrates metabolism (Table S3). Bacteria with core genes at D0 were *Lc. lactis*, *Leuc. carnosum*, and *Leuc. citreum* flanked by *W. koreensis*. While the fermentation proceeded, the species contribution changed in favor of *S. malefermentans*, *Leuc. citreum*, and *P. parvulus*. Although for few days (from D3 to D7), *L. sakei* also contributed to the core genes. The dendrogram based on the coverage frequency of the annotated genes was generated using distance Bray-Curtis (Fig. 4C). The whole fermentation process was clearly divided into three main clusters according to the time points, which identify three phases (from D0 to D1, D2 to D14, and D21 to D42) of fermentation. As indicated by KEGG orthologs (KO) the functional genes capacity of sauerkraut samples was different during the fermentation. The linear discriminant analysis (LDA) scores identified four KEGG orthologs (KO) with significant coverage after 1 week of fermentation (Class B). The KEGG orthologs K00001 (alcohol dehydrogenase [EC:1.1.1.1]), K00796 (dihydropteroate synthase [EC:2.5.1.15]), K02755 (beta-glucoside PTS system EIIA component [EC:2.7.1.-]) and K06147 (ATP-binding cassette, subfamily B, bacterial) were related to carbohydrate metabolism, cofactors metabolism, and signaling and cellular processes, respectively.

### Annotated genes correlated with phenolic compounds.

The evaluation of genes involved in phenolic compounds metabolism was carried out by using two strategies. At first, by checking the presence of these genes in the complete MAGs, and second, in the scaffolds assigned by taxonomy from a global metadata table. The second strategy was used to increase the possibility of identifying the presence of these genes in the species/MAGs assigned with only assembled and annotated fragments of DNA. Among annotated genes linked to the phenolic compounds metabolism, *padA*, *hcrAB*, *par1*, and *hcrF* were not found in MAGs. However, the core species such as *S. malefermentans*, *Lc. lactis*, *P. parvulus*, and *Leuc. citreum* had *VprA* annotated gene (Fig. S3A), while *padR* was annotated *in S. malefermentans*, *L. sakei*, *Lc. lactis*, *L. piscium*, *L. carnosum*, *Leuc. citreum*, *Leuc. gelidum*, *P. parvulus*, and *W. koreensis* (Fig. S3B).

### Correlations among microbiome composition, physicochemical, and biochemical parameters and phenolics profile.

**FIG 5:**
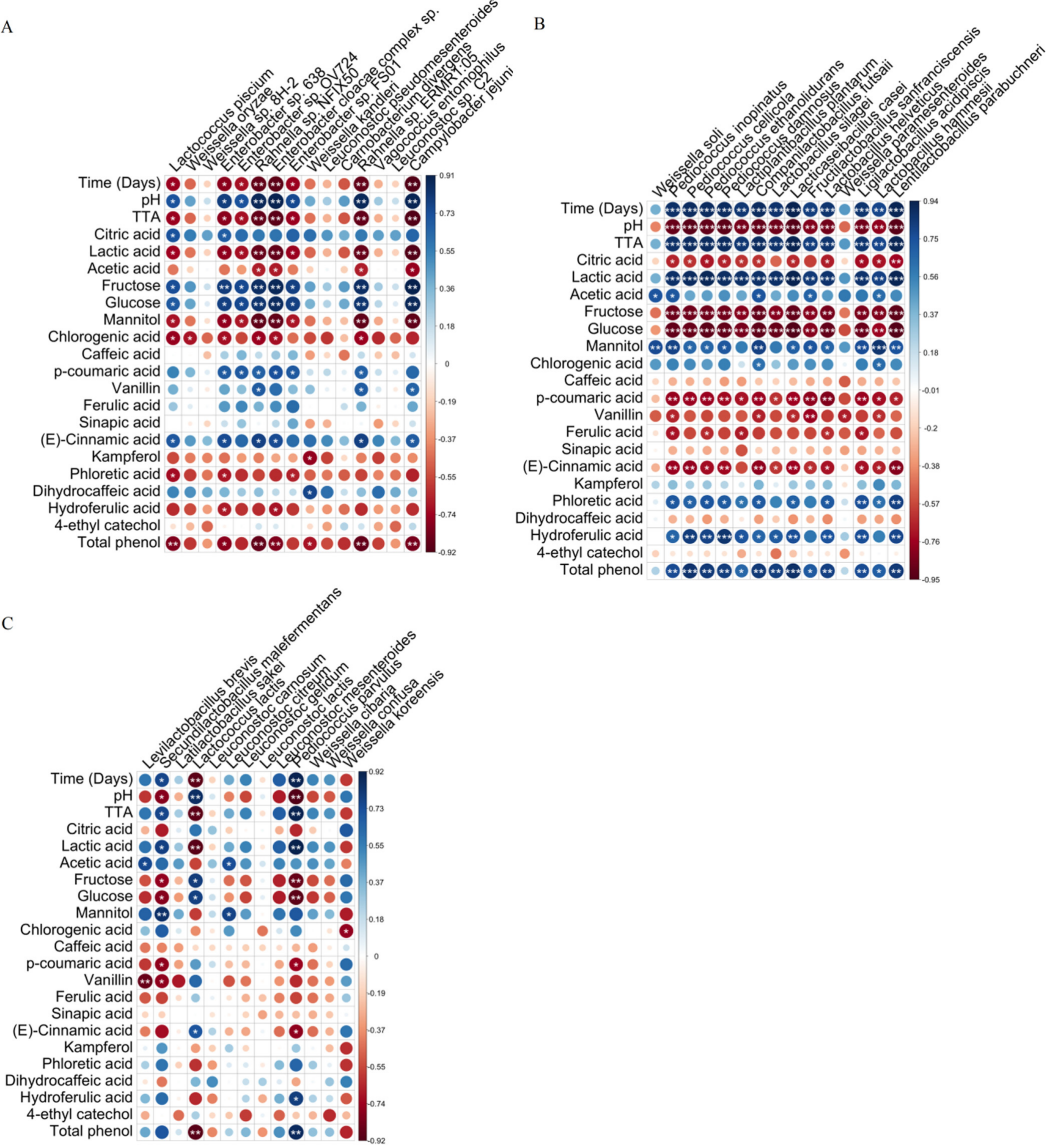
Graphical representation of correlations between sauerkraut microbiome and time of fermentation (days), physicochemical and biochemical composition, and phenolic compounds profile. Spearman's rank correlation matrix between the top 15 bacterial species identified during the first week of fermentation (A), the top 15 species dominant identified between 2 weeks and the end of fermentation (B), overall sauerkraut microbiome throughout the fermentation having a minimum presence threshold of 85% (C). To assess the association between continuous and dichotomous variables, a point-biserial correlation was used. Large and small circles indicate strong and weak correlations, respectively. Colors of the scale bar describe the type of correlation: 1 indicates a perfect positive correlation (dark blue) and −1 indicates a perfect negative correlation (dark red) between two microbial populations. The significance *P* values corrected by FDR are represented by *, < 0.05; **, < 0.01; ***, < 0.001.

Correlation matrixes were established throughout fermentation based on spearman correlation coefficients (Fig. 5). The first correlation was with the top 15 bacterial species identified during the first 7 days of fermentation (Fig. 5A). Then with top 15 bacterial species found between 14 and 42 days of fermentation (Fig. 5B), and finally the overall sauerkraut microbiome throughout the fermentation having a minimum presence threshold of 85% (Fig. 5C). A different (*P* < 0.05) bacterial species contribute was found as determined through the correlation coefficient and *P*-value (Tables S4 to S6). At the beginning of the fermentation, Enterobacter sp., *Rahnella* sp., *Lc. piscium*, Campylobacter jejuni, and Weissella kandleri were negatively correlated with TTA, lactic and acetic acids, mannitol, chlorogenic acid, kaempferol, and phloretic and dihydroferulic acids, while they positively correlated with citric acid, fructose, glucose, *p*-coumaric acid, vanillin, ferulic acid, (E)-cinnamic acid, and dihyrocaffeic acid (Fig. 5A). Subsequently, *Pediococcus* species and lactobacilli were predominant, and showed a negative correlation with pH, citric acid, fructose, glucose, *p*-coumaric acid, vanillin, ferulic, and (E)-cinnamic acids. They were the main contributors to the increase in total free phenolics content, and positively correlated with lactic, acetic, chlorogenic, phloretic, and dihydroferulic acids (Fig. 5B). Considering the whole fermentation time, lactobacilli, *Leuconostoc*, and *Weissella* genera as well the two species *Lc. lactis* and *P. parvulus* were the main microorganisms harboring the sauerkrauts (data not shown). Besides, *S. malefermentans* and *P. parvulus* had a strong positive co-occurrence (rho > 0.65; FDR < 0.05), whereas a negative correlation was found between *Lc. lactis* and *W. koreensis* (rho < −0.65; FDR <0.05) (data not showed). *Leuc. citreum* and *Leuc. mesenteroides* positively correlated with acetic acid and mannitol, while negatively correlated with fructose. *L. plantarum* positively correlated with pH, lactic acid, total free phenolics, *p*-coumaric, ferulic, phloretic, and dihydroferulic acids (Fig. 5C).

## DISCUSSION

While having well established the microbial succession driving spontaneous sauerkraut fermentation (8), the resulting microbiome functionalities and their impact on the biochemical changes need further investigation. The approach we adopted in this study allowed an insight on how secondary plant metabolites, microbial metabolisms, and microbiomes shaping and assembly are associated, with a focus on phenolics and neglected LAB species. Under the condition of our study, culturing revealed the succession of different LAB during sauerkrauts processing, and allowed to identify *Leuc. citreum* and *Leuc. mesenteriodes* as the species responsible to start the fermentation, which were gradually replaced by *P. parvulus*, while *L. plantarum* became the dominant species at the end of fermentation. These dynamics reflected the typical microbial succession of sauerkraut fermentation (22, 23). Metagenomic approach (shotgun) revealed how the core genus microbiome mainly comprises *Pediococcus* sp., *Leuconostoc* sp., and lactobacilli at the beginning of fermentation. The late stage of fermentation was dominated by *Lactococcus* sp. and lactobacilli as determined through co-occurrence patterns (8, 9, 24–28). The high level of chloroplast DNA might reduce the coverage of the microorganism and thus the number of complete MAGs, although countermeasures with satisfactory results were applied. On the other hand, culture-dependent approaches may fail to detect viable but not easily cultivable cells. Currently, the best choice is still to combine the two above approaches. In contrast to what we found by culturing, *S. malefermentans* was identified throughout the fermentation by the metagenomic approach, becoming a species of the core microbiome especially during the last weeks of fermentation. To the best of our knowledge, no similar data were found until now. *S. malefermantans* is a heterofermentative LAB, able to grow both at 15°C (temperature used for sauerkraut fermentation) and 30°C, and glucose is its main carbon source (29). The potentiality of this species has not yet fully explored, but based on our results it is arguable that *S. malefermentans* might have a key role during sauerkraut fermentation carried out at low temperature. Thus, we assume that sauerkraut LAB successions can be different depending on the fermentation temperature despite the use of the same raw materials and preparation method. Predicted gene functions and KEGG pathways were comparable with previous findings, in which carbohydrate, amino acid, and nucleotide metabolisms are the main metabolic pathways underlying sauerkraut fermentation (8, 30). Several lactobacilli, which were found in our sauerkraut, have genes encoding dihydropteroate synthase, an essential enzyme, which is involved in metabolic pathway to synthesize dihydrofolate (31). Reduced folate cofactors are required by most of the organisms to synthesize a variety of metabolites and vitamins (32). Furthermore, during the last phase of fermentation, the *Lactobacillaceae* family harbored a high abundance of genes encoding for alcohol dehydrogenase, which is involved in the aroma compounds production. Under our experimental conditions, these genes resulted mostly associated with *S. malefermentans*, although in the past they were mainly attributed to other lactobacilli, like *L. plantarum*, L. brevis, and Limosilactobacillus reuteri (33–35).

Aiming to explain the role of the microbial community on the nutritional features and compounds that can affect the sensory properties of sauerkraut, the Spearman’s correlation analysis was applied between microbiota and metabolites such as sugars, organic acids, and phenolic compounds. This study confirmed that some *Leuconostoc* species are involved in mannitol production, due to negative correlation with fructose and positive correlation with mannitol and acetic acid (36). Lactobacilli and *Pediococcus* species resulted the main producers of lactic acid, as showed by the strong positive correlation, with glucose and fructose as the major fermentable sugars consumed by them (9, 37, 38). The negative correlation between lactobacilli and *Pediococcus* species and phenolic acids was related to their capability to metabolize hydroxycinnamic acids, which are precursors of phloretic and dihydroferulic acids (15, 18, 39). Metabolism of phenolic compounds was recognized as a complementary strategy adopted by LAB for plant adaptation (40), where the physiological significance of phenolic acids metabolism has been explained as the regeneration of reduced cofactors, which in turn increase the energy yield along phosphoketolase pathway (14). Conversion of phenolic acids through phenolic acid decarboxylase and reductase has been previously described in homo- and hetero-fermentative LAB (15, 18, 39). In particular, hydroxycinnamic acids (e.g., *p*-coumaric, caffeic and ferulic acids) may be decarboxylated or reduced to the corresponding vinyl (p-vinylphenol, vinylcatechol, and vinylguaiacol) or reduced derivatives (phloretic, dihydrocaffeic, and dihydroferulic acids) (14, 15). These derivatives exert higher biological and antioxidant activities than their precursors (15, 17, 41) and are linked to the sensory features of fermented foods (42). Confirming the extensive involvement of various species of LAB in the metabolism of phenolic acids, we found that *S. malefermentans*, *L. sakei*, *Lc. lactis*, *L. piscium*, *Leuc. carnosum*, *Leuc. citreum*, *Leuc. gelidum*, *P. parvulus*, and *W. koreensis* species harbored *padR* gene encoding for a phenolic acid decarboxylase transcriptional regulator (43). The core species, including *S. malefermentans*, *Lc. lactis*, *P. parvulus*, and *Leuc. citreum*, were shown also to hold *VprA* gene, which encodes for a vinyl phenol reductase. This enzyme was previously characterized only in *L. plantarum*, and lead to the reduction of vinyl derivatives into ethylphenols (4-ethylphenol, 4-ethylcatechol, and 4-ethylguaiacol) (44). Although at a very low-perception threshold, vinylphenols, and ethylphenols can have a high impact on the aroma of sauerkraut. Based on the information reported in the literature at the pure culture level, we are confident of the presence of additional genes associated with the reduction and decarboxylation of phenolic acids (e.g., *padA*, *hcrAB*, *par1*, and *hcrF*), which were not found under the conditions of our study probably due to the lack of complete MAGs for all species involved (45).

Our results could help to understand which species can be involved in the changes of phenolic compounds during the spontaneous fermentation of sauerkraut besides *L. plantarum* (43). The phenolic compounds profile of raw white cabbage revealed sinapic acid as the most abundant phenolic acid (46), followed by ferulic, chlorogenic, (E)-cinnamic, *p*-coumaric, and caffeic acids. Despite the identification and quantification of many inherent compounds from red and white cabbages (47–49), the knowledge on phenolic compound metabolism occurring during spontaneous sauerkraut fermentation is still scarce. Under our experimental conditions, the metabolism of phenolic acids was consistent mainly during the last part of the sauerkraut fermentation, whereas free phenolic acids increased at the beginning of the fermentation. Such increase is attributable to the ability of LAB to break plant cell wall and release free phenolic acids through microbial esterase and glycosyl hydrolases (50, 51). An additional outcome by microbial metabolism was the increased accessibility of further nonextractable phenolics, like kaempferol, due to the degradation of associated proteins and carbohydrates (52, 53).

### Conclusions.

This study provided a new perspective on the bacterial community succession during sauerkraut fermentation, and on resulting metabolic functions, which are critical to the modern industrial production of fermented foods. While we confirmed the key role of several well-known core microbiome species, we also proposed *P. parvulus* and *S. malefermentans* as novel key players during fermentation at low temperature of sauerkrauts. Certain functions of sauerkraut microbiome have also been overlooked in the past. For the first time, we related changes in the phenolics profile to changes in the microbiome. Annotated genes linked to the phenolics metabolism were found in many core species during the whole process, although the framework presented is still fragmentary. A high metabolic potential for phenolics bioconversion emerged for lactobacilli and *Pediococcus* spp. through correlation analysis between microbiome composition and phenolics profile. Further studies to exploit neglected bacterial players as potential starters should be encouraged to optimize the fermentation processes and to obtain sauerkrauts with improved and standardized nutritional and sensory features.

## MATERIALS AND METHODS

### Plant material and sauerkraut fermentation.

White cabbages cultivated in South Tyrol and commonly used by local sauerkraut producers were provided from the Lechner Kraut company (Lasa, Bolzano), and used for making sauerkrauts at the pilot level. The spontaneous fermentation was carried out according to the traditional procedure adopted by the company (54). White cabbages were chopped into small pieces and put in jars (three jars for each analysis time) containing 1.3% to 1.4% of salt (NaCl), where the cabbage was completely submerged underneath the juice and brine released by squeezing out the small pieces. The spontaneous fermentation was carried out at 15°C for 42 days. Samples were taken at the beginning of fermentation (D0), after D0.5, D1, D2, D3, D4, D5, D7, D14, D21, D28, D35, and D42 of fermentation.

### Physicochemical analysis.

TTA was determined on 10 g of sauerkraut homogenized with 90 mL of distilled water using a Stomacher apparatus (Seward, London, UK), and expressed as the amount (mL) of 0.1 M NaOH to reach a pH of 8.3. The value of pH was measured by a Foodtrode electrode (Hamilton, Bonaduz, Switzerland).

### Carbohydrates and organic acids quantification.

Freeze-dried sauerkraut powder (2 g) was extracted with 20 mL of water/perichloric acid (95:5, v:v). Mixture was sonicated (amplitude 60) using a macro-probe (Vibra-Cell sonicator; Sonic and Materials Inc., Danbury, CT) for 1 min (2 cycles, 30 s/cycle, 5-min interval between cycles) in an ice bath. The suspension was held under stirring conditions at room temperature for 1 h, kept at 4°C overnight, and centrifuged at 10,000 rpm for 10 min. Water-soluble extracts (WSE) were filtered and stored at −20°C until further use. Concentrations of glucose, fructose, and mannitol were determined through a HPLC system Ultimate 3000 (Dionex, Germering, Germany) equipped with a Spherisorb column (Waters, Millford, USA) and a Perkin Elmer 200a refractive index detector (55). Lactic, acetic, and citric acids were determined by a HPLC system Ultimate 3000 (Dionex, Germering, Germany) equipped with an Aminex HPX-87H column (ion exclusion, Bio-Rad) and a UV detector operating at 210 nm (56). Organic acids and sugars standards were purchased from Sigma-Aldrich (Steinheim, Germany).

### Total phenolics quantification and free phenolic compounds analysis.

Total phenolic compounds were determined according to Folin-Ciocalteu method using MWSE (57). MWSE was obtained by mixing two grams of freeze-dried samples with 20 mL of methanol/water solution (70:30, v:v) acidified with hydrochloric acid (0.1%, vol/vol). The use of acidified solvents increases the extraction yield and avoids side reactions. Mixture was sonicated (amplitude 60) using a macro-probe (Vibra-Cell sonicator; Sonic and Materials Inc., Danbury, CT) for 1 min (2 cycles, 30 s/cycle, 5-min interval between cycles) in an ice bath. The suspension was incubated at room temperature for 1 h under stirring conditions. The MWSE recovered by centrifugation (10,000 rpm for 10 min) were used after filtration.

### Liquid chromatography-mass spectrometry analysis of phenolic compounds.

Liquid chromatography-mass spectrometry (LC-MS/MS) analysis of free phenolic compounds from MWSE was performed using a UHPLC Dionex 3000 (Thermo Fisher Scientific, Germany), coupled to a TSQ Quantum Access MAX Triple Quadrupole mass spectrometer (Thermo Fisher Scientific, Germany), equipped with an electrospray source. Separation of the phenolic compounds was with a Waters Acquity HSS T3 column (1.8 μm, 100 mm × 2.1 mm) (Milford, MA, USA), kept at 40°C. Mobile phase A was water containing 0.1% formic acid; mobile phase B was acetonitrile containing 0.1% formic acid (51). The flow was 0.4 mL/min, and the gradient profile was: 0 min, 2% B; from 0 to 3 min, linear gradient to 20% B; from 3 to 4.3 min, isocratic 20% B; from 4.3 to 9 min, linear gradient to 45% B; from 9 to 11 min, linear gradient to 100% B; from 11 to 13 min, wash at 100% B; from 13.01 to 15 min, back to the initial conditions of 5% B. The injection volume was 3 μL. Calibration curves were determined with selected chemical standards and data expressed as μg g^−1^ of DM, after normalization with the internal standard phloridzin. Target phenolic compounds were detectable under multiple reaction monitoring (MRM) mode and the compounds were identified based on their reference standard, retention time, qualifier and quantifier ion. The management of the chromatographic system and data acquisition was by Xcalibur software version 4.1 (Thermo Fisher Scientific, Germany).

### Identification and quantification of phenolic acid derivatives.

During the optimization step, the four phenolic acid derivatives were successfully infused into the TSQ Quantum Access MAX Triple Quadrupole mass spectrometer (Thermo Fisher Scientific), in which the respective parent ions and product fragments were obtained. Nevertheless, the use of Waters Acquity HSS T3 column was unsuitable to detect the phenolic acid derivatives. Therefore, separation, determination, and quantification of these derivatives from MWSE were performed by using an HPLC system Ultimate 3000 (Dionex, Germering, Germany). HPLC system was equipped with a Kinetex C18 Phenomenex (150 mm × 4.6 mm with a particle size of 5 μm) column (Thermo Fisher Scientific), a photodiode array detector (PAD 3000), low-pressure pump Ultimate 3000, and an injector loop Rheodyne (Rheodyne, USA, volume 20 μL), according to the method validated by Filannino (58).

### Microbiological analysis and LAB isolation.

Ten grams of sauerkraut were suspended (1:10 vol/vol) in sterile 0.9% (wt/vol) sodium chloride solution and homogenized. Mesophilic LAB and yeasts were determined on MRS agar (Oxoid Ltd., Basingstoke, Hampshire, UK), containing 0.1% of cycloheximide (Sigma Chemical Co.), at 30°C for 48 h and 72 h under anaerobic conditions, and on MEA (Oxoid), added of 150 ppm chloramphenicol, at 25°C for 72 h, respectively. The enumeration of total mesophilic bacteria was on PCA agar (Oxoid) at 30°C for 48 h. Gram-positive, catalase-negative, nonmotile rod, and cocci isolates were cultivated onto MRS broth (Oxoid Ltd.) at 30°C for 24 h under anaerobic condition, then restreaked onto the agar medium (8, 10). This procedure was repeated three times to ensure pure cultures.

### Genotyping and identification of LAB isolates.

For the extraction of genomic DNA, 2 mL of MRS culture broth from each purified isolate were used. Two primer pairs LacbF/LacbR and LpCoF/LpCoR (Sigma Chemical Co. Milan, Italy) were used to amplify the 16S rRNA gene of LAB (21). Eurofins Genomics (Germany) carried out the sequencing of PCR products. Sequence comparison was against the National Center for Biotechnology Information (NCBI) genomic database with BLAST (http://blast.ncbi.nlm.nih.gov/Blast.cgi) search alignment tool. Differentiation between *Lactiplantibacillus* spp. and *Lacticaseibacillus* spp. was carried out according to Torriani (59) and Ward and Timmins (60), respectively.

Randomly amplified polymorphic DNA-PCR (RAPD-PCR) was carried out using three arbitrary primers: M13, P7, and P4 (Invitrogen Life Technologies, Milan, Italy) for genotyping of bacterial isolates (61).

### Total microbial DNA extraction and bacterial metagenome sequencing.

Sauerkraut samples were subjected to DNA extraction using the SPIN Kit for Soil (MP Biomedicals, Italy), according to the manufacturer’s instruction. Extracted DNA was stored at −20°C. The assessment of the DNA concentration was with the fluorimeter Qubit 2.0 (Invitrogen, Italy). DNA processing was performed at the Genomics Platform–Unit of Computational Biology (Edmund Mach Foundation, TR, Italy), DNA purification, library preparation, and sequencing Illumina Novaseq 6000 platform, paired-end strategy, following standard protocols. Shotgun metagenome sequencing resulted in 3.56 to 6.04 Gbp, depending on time point of sauerkraut collection for a total 205,940,000 paired-end reads. Trimmomatic (v0.39.1) (62) was used for quality filtering, using parameters LEADING:20 TRAILING:20 SLIDINGWINDOW:4:20 MINLEN:70,” adapters and sequences derived from phi X 174 were removed using BBtools, while pair-end sequence reads were merged with BBMerge (version released Nov 2016). Approximately, 6% of the reads were dropped during quality check and 1.5% were found to contain adapters or phi X 174. Brassica genomic DNA was identified and removed using by aligning reads on genome (assembly version ARS-UCD1.2) with bowtie 2 (v2.3.4.3) and using SAMtools (v1.9) to convert and sort the “sam” files. The number of contaminants reads identified was at maximum 4.6%. Only high-quality reads were further used to assemble the metagenome using MEGAHIT (v1.2.4-beta) using standard parameters (63). Realignment was performed to determine the scaffolds coverage with bowtie 2 (v2.3.4.3) (64), and “sam” files were converted to “bam,” sorted and indexed using SAMtools package (v1.9) (65). Metabat2 software (v2.12.1) (66) were used for binning with default parameters and using the sorted bam files previously generated. MAGs completeness and contamination were predicted with checkM (v1.0.7) (67). The same software was used to calculate the MAGs and scaffolds relative abundance, taking into consideration the coverage values and scaffolds coverage obtained from bam files. Clustering of the MAGs coverage values was performed with Pearson correlation using MeV software based on the relative abundance of each MAG (68). Prodigal (V2.6.3: February, 2016) was used in metagenome mode to predict protein-encoding genes in the scaffolds and in “normal mode” to predict genes in the MAGs (69). The annotated KOs were further used by KEGG mapper to directly link to phenotypes and other higher functional traits (70). KEGG and its internal tool BlastKoala with standard parameters, and CARD with the same settings as described above were used to predict the ARGs in high-quality MAGs. Taxonomic assignment of the scaffolds was done with CAT/BAT tool (v4.6) (71) while taxonomic assignment of the MAGs was performed using GTDB-Tk (v0.3.2).

### Statistical analysis.

Each analysis was performed in duplicate on three biological replicates. Data of physicochemical and biochemical analysis were subjected to one-way ANOVA with Tukey’s test (*P* < 0.05) using the statistical software Statistica for Windows (Statistica 7.0 per Windows). Statistical analyses of the microbiome were performed with R software (version 3.4.4) using multiple R packages: phyloseq (version 1.24.0) to facilitate the import, storage, analysis, and graphical display of microbiome census data (72); a filter was applied following at least 20% of its values should contain at least four counts. Data analyses were normalized by total sum scaling (TSS) normalization method. The stacked area plot, PCA, and principal coordinate analysis (PCoA) were carried out by ggplot 2 Wickham (73). A hierarchical clustering using Bray-Curtis dissimilarity matrix of the gene annotated by KEGG was performed using the hclust tool (R package, https://www.rdocumentation.org/packages/stats/versions/3.6.1/topics/hclust). The LDA was used to discover the KO discriminating sauerkraut after 7 days and after 14 days of fermentation (74). Spearman’s rank correlation matrix, *P* values, and false discovery rate correction (FDR) were generated by cor.test and visualized by corrplot package (75).

### Data availability.

Data sets are publicly available at NCBI under the accession number (PRJNA761016).
