# Supplementary material for: How Microbiome Composition Correlates with Biochemical Changes during Sauerkraut Fermentation: a Focus on Neglected Bacterial Players and Functionalities
Source: Microbiol Spectr. 2022 Jun 14;10(4):e00168-22. doi: 10.1128/spectrum.00168-22 (PMC9430578; doi:10.1128/spectrum.00168-22)
Supplement: Supplemental file 1 — Supplemental material. Download spectrum.00168-22-s0001.pdf, PDF file, 0.7 MB [file spectrum.00168-22-s0001.pdf]

Table S1. Numbers of genes associated to carbohydrates metabolism for each species identified during spontaneous fermentation of sauerkraut carried out at 15 ºC for 42 days. Samples were taken at the beginning of fermentation (D0), after 0.5 (D0.5), 1 (D1), 2 (D2), 3 (D3), 4 (D4), 5 (D5), 7 (D7), 14 (D14), 21 (D21), 28 (D28), 35 (D35), and 42 (D42) days of fermentation.

Carbohydrate metabolism	D0	D0.5	D1	2D	3D	4D	5D	7D	14D	21D	28D	35D	42D
Lactococcus lactis	253	253	253	253	253	253	16	16	44	16	16	33	0
Leuconostoc carnosum	194	170	150	194	194	194	32	27	27	27	27	25	27
Leuconostoc citreum	163	85	186	192	197	194	194	194	194	192	194	191	194
Weissella koreensis	137	137	61	137	137	137	6	0	6	6	6	6	6
Secundilactobacillus malefermentans	83	49	50	62	153	153	158	158	159	158	158	153	158
Pediococcus parvulus	40	44	33	25	52	45	54	53	53	54	54	54	54
Lactococcus piscium	34	34	11	189	238	238	0	0	0	0	0	0	0
Enterobacter sp. OV724	15	15	15	14	0	15	0	0	0	0	0	0	0
Latilactobacillus sakei	15	0	0	14	169	167	138	173	8	9	12	11	8
Spermatophyta	5	5	5	5	5	5	5	5	5	5	5	5	5
Rahnella sp. NFIX50	2	2	2	2	2	2	0	0	0	0	0	0	0
Weissella cibaria	2	2	2	2	2	28	1	0	1	1	1	1	1
Leclercia sp. W6	1	1	1	1	0	1	0	0	0	0	0	0	0
Pantoea agglomerans*	1	1	1	1	0	0	0	0	0	0	0	0	0
Rahnella sp. NRRL B-41462	1	1	1	1	1	1	0	0	0	0	0	0	0
Levilactobacillus brevis	0	0	0	0	2	2	2	2	2	2	2	2	2
Latilactobacillus curvatus	0	0	0	0	0	0	1	0	0	0	1	0	0
Companilactobacillus futsaii	0	0	0	0	0	0	1	0	1	0	1	1	0
Lentilactobacillus parafarraginis	0	0	0	0	0	0	0	0	2	0	0	0	0

Lactiplantibacillus plantarum	0	0	0	0	6	6	6	6	6	6	6	6	6
Secundilactobacillus silagei	0	0	0	0	19	1	27	18	18	27	26	27	23
Leuconostoc gelidum	0	1	0	1	65	2	0	1	0	0	0	0	0
Leuconostoc mesenteroides	0	0	0	4	9	4	1	1	1	1	1	1	1
Leuconostocaceae bacterium	0	0	3	0	3	3	3	3	3	0	3	3	3
Pediococcus cellicola	0	0	0	0	4	0	4	4	4	4	4	4	4
Pediococcus damnosus	0	0	0	0	3	0	3	3	3	3	3	3	3
Pediococcus ethanolidurans	0	0	0	0	10	0	10	10	12	12	12	12	10
unclassified Lactobacillus	0	0	0	0	3	0	3	4	4	4	4	3	4
Weissella confusa	0	0	0	0	1	0	0	0	0	0	1	0	0
Weissella kandleri	0	1	0	1	1	1	0	0	0	0	0	0	0
Weissella paramesenteroides	0	0	0	0	2	2	0	0	0	0	0	0	0
**Total**	946	801	774	1098	1531	1454	665	678	553	527	537	541	509

Table S2. Binning parameters of the 9 high quality genomes MAGs (Metagenome-Assembled-Genomes) after binning and *de-novo* reassembly of all the metagenome sequences from sauerkraut samples collected at the beginning of fermentation (D0), after 0.5 (D0.5), 1 (D1), 2 (D2), 3 (D3), 4 (D4), 5 (D5), 7 (D7), 14 (D14), 21 (D21), 28 (D28), 35 (D35), and 42 (D42) days of fermentation.

**MAG ID**	**MAG name**	**Completeness**	**Contamination**	**Strain heterogeneity**	**MAG size (Mbp)**	**0D**	**0.5D**	**1D**	**2D**	**3D**	**4D**	**5D**	**7D**	**14D**	**21D**	**28D**	**35D**	**42D**
**MAG_22**	*Pediococcus parvulus MAG_22*	97.22	0.87	60	1.933514	0.009938	0.011321	0.002125	0.001582	0.421514	0.01677	4.484935	38.06915	53.15972	84.53077	62.1168	91.87508	56.48383
**MAG_23**	*Lactococcus lactis MAG_23*	90.91	0.88	33.33	2.086278	0.40896	0.688253	0.20813	0.262873	0.340631	0.003504	0	0	7.13E-06	0	0	2.44E-05	1.04E-05
**MAG_43**	*Weissella confusa MAG_43*	89.26	0.31	100	1.950252	0.005422	0.000823	0.000207	0.000206	15.08355	6.20135	0.001319	0.002259	0.003172	0.003893	0.003399	0.005441	0.002523
**MAG_29**	*Erwinia persicina MAG_29*	87.24	0.38	16.67	4.273701	0.002672	0.004671	0.003311	0.003628	0.506218	0.149372	0.509917	0.065694	0.008023	0.000782	0.000425	0.001737	0.000797
**MAG_36**	*Leuconostoc sp. MAG_36*	85.64	8.25	32.43	2.68753	0.001079	0.000295	6.53E-05	5.39E-05	0.523913	0.578245	0.000361	0.000257	0.000155	0	0.000222	0	1.61E-05
**MAG_27**	*Lactococcus piscium_C MAG_27*	85.51	4.81	70	2.427631	0.000763	0.00022	5.06E-05	0.01918	0.477859	0.542194	0	0	0	0	0	0	0
**MAG_18**	*Raoultella terrigena MAG_18*	75.16	5.26	100	4.990814	19.65899	19.23193	19.98495	20.71456	0.040932	0.230133	0.004003	0.002811	0.0021	1.85E-05	5.2E-06	1.7E-05	4.33E-06
**MAG_9**	*Leuconostoc mesenteroides MAG_9*	68.42	0	0	1.134091	0.000798	0.000302	0.000279	0.067051	2.561295	0.011401	1.41317	0.051756	0.333348	1.193912	6.962449	0.649091	8.712914

**MAG_32**	*Secundilactobacillus G malefermentans MAG_32*	66.82	0.16	0	1.58296	0.00078	0.00023	9.98E-05	0.000102	0.018371	0.009059	0.3593	3.862364	3.593749	4.19656	2.645824	1.982934	1.344819

Table S3. Numbers of genes associated to lipid metabolism for each species identified during spontaneous fermentation of sauerkraut carried out at 15 ºC for 42 days. Samples were taken at the beginning of fermentation (D0), after 0.5 (D0.5), 1 (D1), 2 (D2), 3 (D3), 4 (D4), 5 (D5), 7 (D7), 14 (D14), 21 (D21), 28 (D28), 35 (D35), and 42 (D42) days of fermentation.

**Lipid metabolism**	**0D**	**0.5D**	**1D**	**2D**	**3D**	**4D**	**5D**	**7D**	**14D**	**21D**	**28D**	**35D**	**42D**
Lactococcus lactis	253	253	253	253	253	253	16	16	44	16	16	33	0
Leuconostoc carnosum	194	170	150	194	194	194	32	27	27	27	27	25	27
Leuconostoc citreum	163	85	186	192	197	194	194	194	194	192	194	191	194
Weissella koreensis	137	137	61	137	137	137	6	0	6	6	6	6	6
Secundilactobacillus malefermentans	83	49	50	62	153	153	158	158	159	158	158	153	158
Pediococcus parvulus	40	44	33	25	52	45	54	53	53	54	54	54	54
Lactococcus piscium	34	34	11	189	238	238	0	0	0	0	0	0	0
Enterobacter sp. OV724	15	15	15	14	0	15	0	0	0	0	0	0	0
Latilactobacillus sakei	15	0	0	14	169	167	138	173	8	9	12	11	8
Spermatophyta	5	5	5	5	5	5	5	5	5	5	5	5	5
Rahnella sp. NFIX50	2	2	2	2	2	2	0	0	0	0	0	0	0
Weissella cibaria	2	2	2	2	2	28	1	0	1	1	1	1	1
Leclercia sp. W6	1	1	1	1	0	1	0	0	0	0	0	0	0
Pantoea agglomerans	1	1	1	1	0	0	0	0	0	0	0	0	0
Rahnella sp. NRRL B-41462	1	1	1	1	1	1	0	0	0	0	0	0	0
Levilactobacillus brevis	0	0	0	0	2	2	2	2	2	2	2	2	2
Latilactobacillus curvatus	0	0	0	0	0	0	1	0	0	0	1	0	0
Companilactobacillus futsaii	0	0	0	0	0	0	1	0	1	0	1	1	0
Lentilactobacillus parafarraginis	0	0	0	0	0	0	0	0	2	0	0	0	0

Lactiplantibacillus plantarum	0	0	0	0	6	6	6	6	6	6	6	6	6
Secundilactobacillus silagei	0	0	0	0	19	1	27	18	18	27	26	27	23
Leuconostoc gelidum	0	1	0	1	65	2	0	1	0	0	0	0	0
Leuconostoc mesenteroides	0	0	0	4	9	4	1	1	1	1	1	1	1
Leuconostocaceae bacterium	0	0	3	0	3	3	3	3	3	0	3	3	3
Pediococcus cellicola	0	0	0	0	4	0	4	4	4	4	4	4	4
Pediococcus damnosus	0	0	0	0	3	0	3	3	3	3	3	3	3
Pediococcus ethanolidurans	0	0	0	0	10	0	10	10	12	12	12	12	10
unclassified Lactobacillus	0	0	0	0	3	0	3	4	4	4	4	3	4
Weissella confusa	0	0	0	0	1	0	0	0	0	0	1	0	0
Weissella kandleri	0	1	0	1	1	1	0	0	0	0	0	0	0
Weissella paramesenteroides	0	0	0	0	2	2	0	0	0	0	0	0	0
**Total**	946	801	774	1098	1531	1454	665	678	553	527	537	541	509

Table S4. P values corrected by False Discovery Rate (FDR) of the correlations between the top 15 bacterial species present at the beginning of fermentation (from D0 to D7) and time of fermentation (days), physico-chemical and biochemical composition, and phenolic compounds at the same interval of time.

	Lactococcus piscium	*Weissella oryzae*	*Weissella* sp. 8H-2	*Enterobacter* sp. 638	*Enterobacter* sp. OV724	*Rahnella* sp. NFIX50	*Enterobacter cloacae complex*	*Enterobacter* sp. FS01	*Weissella kandleri*	*Leuconostoc pseudomesenteroides*	*Carnobacterium divergens*	*Rahnella* sp. ERMR 1:05	*Vagococcus entomophilus*	*Leuconostoc* sp. C2	*Campylobacter jejuni*
**Time (Days)**	0.023	0.216	0.667	0.014	0.033	0.002	0.002	0.023	0.249	0.481	0.163	0.002	0.475	0.667	0.001
**pH**	0.029	0.249	0.667	0.011	0.026	0.002	0.001	0.019	0.282	0.481	0.240	0.002	0.475	0.667	0.002
**TTA**	0.023	0.216	0.667	0.014	0.033	0.002	0.002	0.023	0.249	0.481	0.163	0.002	0.475	0.667	0.001
**Citric acid**	0.029	0.117	0.249	0.041	0.074	0.113	0.087	0.051	0.083	0.215	0.288	0.113	0.249	0.249	0.058
**Lactic acid**	0.024	0.249	0.667	0.015	0.035	0.002	0.002	0.022	0.214	0.481	0.174	0.002	0.475	0.667	0.001
**Acetic acid**	0.255	0.678	0.958	0.249	0.375	0.044	0.041	0.278	0.667	0.941	0.651	0.044	0.973	0.958	0.020
**Fructose**	0.022	0.214	0.678	0.009	0.018	0.002	0.002	0.012	0.249	0.475	0.237	0.002	0.481	0.678	0.001
**Glucose**	0.037	0.255	0.799	0.013	0.018	0.005	0.002	0.012	0.319	0.568	0.285	0.005	0.599	0.799	0.002
**Mannitol**	0.036	0.254	0.760	0.018	0.037	0.002	0.002	0.037	0.248	0.475	0.568	0.002	0.448	0.760	0.002
**Chlorogenic acid**	0.021	0.037	0.215	0.033	0.123	0.022	0.035	0.234	0.123	0.077	0.565	0.022	0.062	0.215	0.058
**Caffeic acid**	0.975	0.973	0.568	0.534	0.327	0.646	0.448	0.350	0.389	0.694	0.249	0.646	0.618	0.568	0.726
***p*-coumaric acid**	0.140	0.333	0.808	0.039	0.037	0.047	0.024	0.037	0.668	0.626	0.808	0.047	0.667	0.808	0.060
**Vanillin**	0.300	0.797	0.853	0.264	0.389	0.037	0.075	0.380	0.394	0.872	0.916	0.037	0.749	0.853	0.047
**Ferulic acid**	0.448	0.817	0.958	0.221	0.153	0.319	0.179	0.069	0.974	0.990	0.665	0.319	0.906	0.958	0.271
**Sinapic acid**	0.988	0.878	0.694	0.668	0.434	0.932	0.608	0.271	0.389	0.488	0.973	0.932	0.375	0.694	0.688
**(E)-Cinnamic acid**	0.033	0.225	0.373	0.021	0.062	0.012	0.021	0.072	0.074	0.170	0.375	0.012	0.153	0.373	0.037
**Kampferol**	0.123	0.275	0.288	0.245	0.375	0.278	0.429	0.427	0.018	0.111	0.713	0.278	0.105	0.288	0.323
**Phloretic acid**	0.031	0.094	0.249	0.039	0.072	0.109	0.066	0.047	0.194	0.255	0.181	0.109	0.288	0.249	0.051
**Dihydrocaffeic acid**	0.151	0.194	0.288	0.327	0.481	0.394	0.630	0.646	0.017	0.077	0.667	0.394	0.074	0.288	0.433
**Dihydroferulic acid**	0.066	0.123	0.393	0.041	0.073	0.066	0.037	0.080	0.493	0.356	0.240	0.066	0.375	0.393	0.072
**4-ethyl catechol**	0.777	0.538	0.215	0.929	0.973	0.694	0.791	0.973	0.749	0.394	0.565	0.694	0.429	0.215	0.630
**Total phenol**	0.009	0.083	0.357	0.014	0.060	0.002	0.010	0.084	0.037	0.131	0.069	0.002	0.105	0.357	0.006

Table S5. P values corrected by False Discovery Rate (FDR) of the correlations between the top 15 bacterial species identified from 7 to 14 days, and time of fermentation (days), physico-chemical and biochemical composition, and phenolic compounds at the same interval of time.

	Weissella soli	***Pediococcus inopinatus***	***Pediococcus cellicola***	***Pediococcus ethanolidurans***	***Pediococcus damnosus***	***Lactiplantibacillus plantarum***	***Companilactobacillus futsaii***	***Secundilactobacillus silagei***	***Lacticaseibacillus casei group***	***Fructilactobacillus sanfranciscensis***	***Lactobacillus helveticus***	***Weissella paramesenteroides***	***Ligilactobacillus acidipiscis***	***Levilactobacillus hammesii***	***Lentilactobacillus parabuchneri***
**Time (Days)**	0.2315	0.0005	0.0003	0.0003	0.0003	0.0011	0.0003	0.0006	0.0001	0.0020	0.0008	0.1398	0.0008	0.0039	0.0001
**pH**	0.2215	0.0004	0.0002	0.0003	0.0002	0.0011	0.0003	0.0006	0.0001	0.0020	0.0008	0.1398	0.0008	0.0039	0.0001
**TTA**	0.2315	0.0005	0.0003	0.0003	0.0003	0.0011	0.0003	0.0006	0.0001	0.0020	0.0008	0.1398	0.0008	0.0039	0.0001
**Citric acid**	0.6047	0.0258	0.0374	0.0146	0.0258	0.0397	0.0364	0.1297	0.0325	0.0559	0.0204	0.6284	0.0101	0.0130	0.0078
**Lactic acid**	0.2291	0.0006	0.0003	0.0002	0.0004	0.0006	0.0005	0.0005	0.0001	0.0015	0.0007	0.1356	0.0007	0.0027	0.0002
**Acetic acid**	0.0101	0.0118	0.1957	0.1568	0.1142	0.1962	0.0101	0.2062	0.1060	0.0213	0.1398	0.0559	0.0577	0.0353	0.0964
**Fructose**	0.1611	0.0006	0.0007	0.0007	0.0008	0.0025	0.0003	0.0025	0.0003	0.0028	0.0011	0.1750	0.0014	0.0020	0.0002
**Glucose**	0.2792	0.0003	0.0005	0.0002	0.0003	0.0010	0.0003	0.0004	0.0001	0.0025	0.0003	0.0712	0.0005	0.0070	0.0001
**Mannitol**	0.0056	0.0030	0.0263	0.0268	0.0125	0.0676	0.0025	0.0648	0.0204	0.0101	0.0255	0.1406	0.0066	0.0008	0.0095
**Chlorogenic acid**	0.1406	0.0755	0.1005	0.1445	0.0712	0.6190	0.0422	0.3657	0.1452	0.1142	0.1047	0.8450	0.0708	0.0334	0.0964
**Caffeic acid**	0.6248	0.4146	0.4111	0.4502	0.3199	0.3132	0.5180	0.5939	0.4582	0.3978	0.3815	0.1063	0.3933	0.3978	0.5160
***p*-coumaric acid**	0.4582	0.0054	0.0066	0.0066	0.0089	0.0130	0.0069	0.0382	0.0075	0.0039	0.0022	0.0748	0.0066	0.0100	0.0228
**Vanillin**	0.1026	0.0254	0.0884	0.0677	0.0768	0.0964	0.0213	0.0712	0.0317	0.0066	0.0519	0.0203	0.0550	0.0353	0.1306
**Ferulic acid**	0.7290	0.0142	0.0701	0.0324	0.0626	0.0172	0.0519	0.0790	0.0760	0.0687	0.0341	0.0964	0.0146	0.1347	0.0760
**Sinapic acid**	0.8990	0.3991	0.4320	0.3853	0.4146	0.0846	0.5831	0.4725	0.5066	0.4223	0.4104	0.3222	0.3579	0.4146	0.4582
**(E)-Cinnamic acid**	0.4146	0.0066	0.0045	0.0109	0.0095	0.0744	0.0037	0.0410	0.0081	0.0334	0.0137	0.5514	0.0118	0.0237	0.0027
**Kampferol**	0.4687	0.3195	0.4276	0.3853	0.4877	0.7160	0.2658	0.7630	0.3871	0.3222	0.3136	0.7613	0.3205	0.0964	0.2774
**Phloretic acid**	0.7685	0.0114	0.0229	0.0101	0.0146	0.0476	0.0130	0.0790	0.0207	0.0658	0.0137	0.6076	0.0070	0.0435	0.0027
**Dihydrocaffeic acid**	0.8688	0.4146	0.4276	0.2952	0.4110	0.7932	0.3301	0.5427	0.2732	0.4502	0.2446	0.8941	0.3275	0.3699	0.2529
**Dihydroferulic acid**	0.9929	0.0245	0.0017	0.0030	0.0008	0.0109	0.0258	0.0146	0.0070	0.0505	0.0095	0.4146	0.0066	0.0712	0.0043
**4-ethyl catechol**	0.5831	0.7613	0.7145	0.6441	0.7579	0.3978	0.7365	0.1737	0.5180	0.7160	0.7637	0.2693	0.8142	0.9452	0.8528
**Total phenol**	0.4643	0.0063	0.0007	0.0012	0.0023	0.0296	0.0016	0.0023	0.0006	0.0128	0.0027	0.5180	0.0055	0.0178	0.0012

Table S6. P values corrected by False Discovery Rate (FDR) of the correlations between all the dominat bacterial species identified throughout the fermentation (from D0 to D42), and time of fermentation (days), physico-chemical and biochemical composition, and phenolic compounds at the same interval of time.

	Levilactobacillus brevis	***Secundilactobacillus malefermentans***	***Latilactobacillus sakei***	***Lactococcus lactis***	***Leuconostoc carnosum***	***Leuconostoc citreum***	***Leuconostoc gelidum***	***Leuconostoc lactis***	***Leuconostoc mesenteroides***	***Pediococcus parvulus***	***Weissella cibaria***	***Weissella confusa***	***Weissella koreensis***
**Time (Days)**	0.1319	0.0235	0.5686	0.0015	0.7402	0.3407	0.1756	0.8153	0.0749	0.0012	0.1925	0.2381	0.1304
**pH**	0.1281	0.0208	0.5466	0.0032	0.7898	0.3407	0.1578	0.8595	0.0739	0.0012	0.1649	0.2105	0.1330
**TTA**	0.1319	0.0235	0.5686	0.0015	0.7402	0.3407	0.1756	0.8153	0.0749	0.0012	0.1925	0.2381	0.1304
**Citric acid**	0.5686	0.0739	0.8508	0.1330	0.5156	0.7104	0.9627	0.9627	0.5657	0.1048	0.7043	0.9627	0.0594
**Lactic acid**	0.1304	0.0203	0.5341	0.0012	0.7402	0.3377	0.1831	0.8608	0.0763	0.0012	0.2086	0.2381	0.1281
**Acetic acid**	0.0263	0.1048	0.2749	0.1460	0.5002	0.0350	0.2425	0.7835	0.1924	0.2425	0.2360	0.3143	0.3407
**Fructose**	0.1909	0.0263	0.5786	0.0160	0.8468	0.2749	0.2005	0.9057	0.0822	0.0032	0.1702	0.2749	0.1048
**Glucose**	0.1084	0.0208	0.4582	0.0203	0.7402	0.4058	0.1509	0.8803	0.0749	0.0015	0.1319	0.2381	0.1330
**Mannitol**	0.0776	0.0036	0.3377	0.1319	0.7092	0.0235	0.2749	0.9627	0.1319	0.0594	0.2979	0.4331	0.0749
**Chlorogenic acid**	0.4582	0.0739	0.8142	0.3835	0.8608	0.2749	0.9927	0.2979	0.7835	0.2105	0.9927	0.8414	0.0263
**Caffeic acid**	0.3143	0.3499	0.4601	0.7835	0.7811	0.7881	0.7143	0.7413	0.8091	0.5813	0.4582	0.8811	0.7479
***p*-coumaric acid**	0.1319	0.0235	0.4859	0.2749	0.7306	0.6187	0.5847	0.9627	0.5147	0.0350	0.2810	0.7402	0.1122
**Vanillin**	0.0032	0.0235	0.0724	0.1048	0.9627	0.1925	0.3091	0.9669	0.4143	0.1048	0.2822	0.4551	0.2749
**Ferulic acid**	0.2105	0.1460	0.7898	0.5341	0.9101	0.8414	0.5686	0.6399	0.3377	0.1509	0.2918	0.5156	0.5248
**Sinapic acid**	0.7440	0.7306	0.9964	0.9854	0.8414	0.9627	0.7402	0.5002	0.5786	0.6188	0.5466	0.6948	0.9300
**(E)-Cinnamic acid**	0.3348	0.0512	0.9057	0.0495	0.6283	0.5684	0.5125	0.9300	0.2749	0.0235	0.2749	0.5684	0.1319
**Kampferol**	0.8899	0.2515	0.9627	0.4582	0.7104	0.5686	0.7440	1.0000	0.9669	0.5403	0.9533	0.6094	0.1122
**Phloretic acid**	0.5813	0.1319	0.7402	0.1161	0.4551	0.8468	0.8360	0.8811	0.4331	0.0965	0.5466	0.8899	0.1161
**Dihydrocaffeic acid**	0.8556	0.3143	0.9854	0.4058	0.2295	0.9627	0.7306	0.5769	0.8468	0.5156	0.9627	0.5248	0.1702
**Dihydroferulic acid**	0.4143	0.1084	0.9339	0.1018	0.3377	0.9669	0.6283	0.5376	0.3780	0.0173	0.5970	0.7043	0.2105
**4-ethyl catechol**	0.5403	0.9778	0.2749	0.5847	0.7835	0.6892	0.1330	0.9214	0.2425	0.6845	0.5722	0.1048	0.5847
**Total phenol**	0.3348	0.0512	0.7765	0.0012	0.3751	0.5847	0.4230	0.4230	0.2314	0.0022	0.5002	0.4941	0.0965

Figure S1. Principal component analysis (PCA) (A) of microbial communities based on Euclidean distance, and Principal coordinate analysis (PCoA) (B) based on Bray-Curtis distance matrix during spontaneous fermentation of sauerkraut at 15 ºC carried out at 15 ºC for 42 days. Samples were taken at the beginning of fermentation (D0), after 0.5 (D0.5), 1 (D1), 2 (D2), 3 (D3), 4 (D4), 5 (D5), 7 (D7), 14 (D14), 21 (D21), 28 (D28), 35 (D35), and 42 (D42) days of fermentation.

A

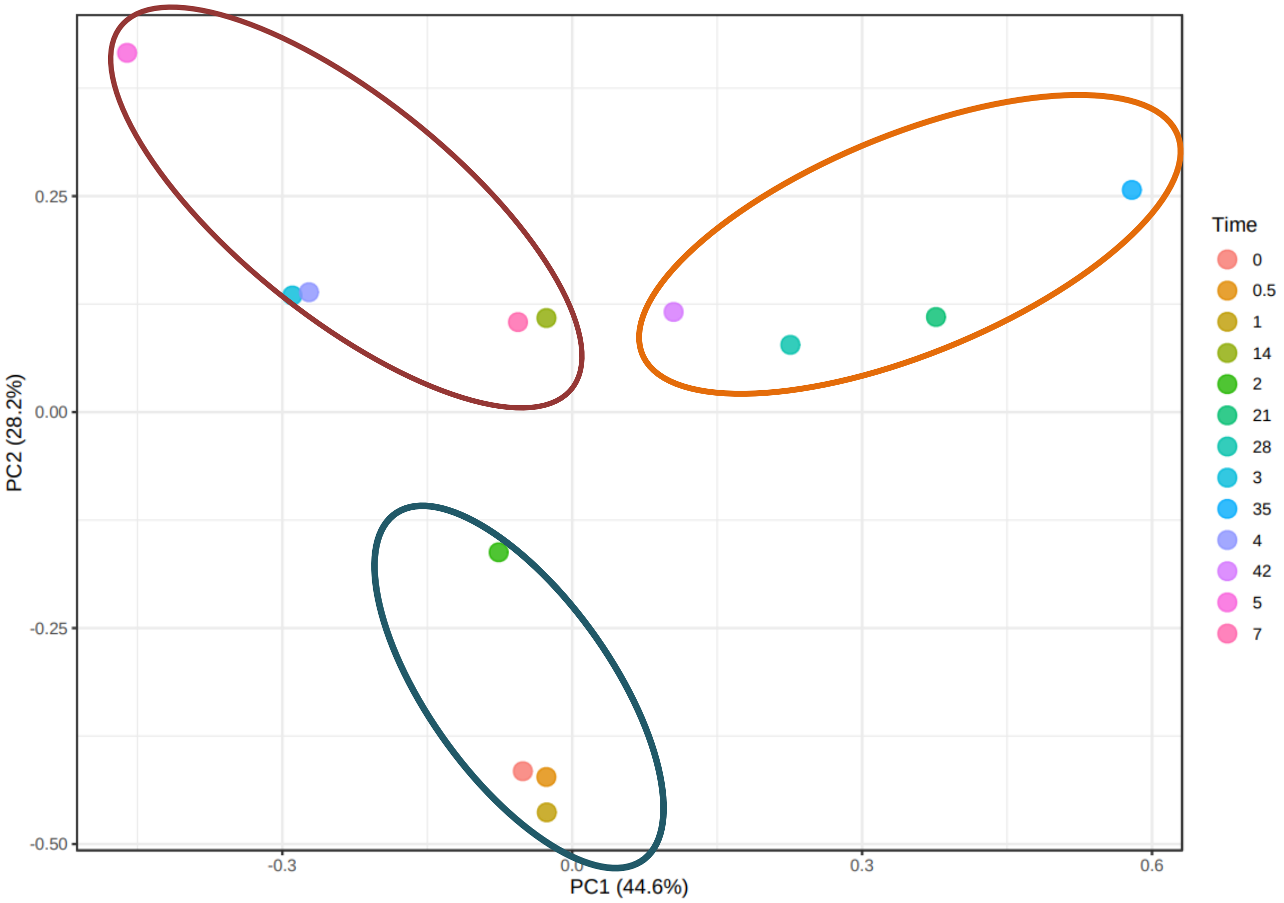

B

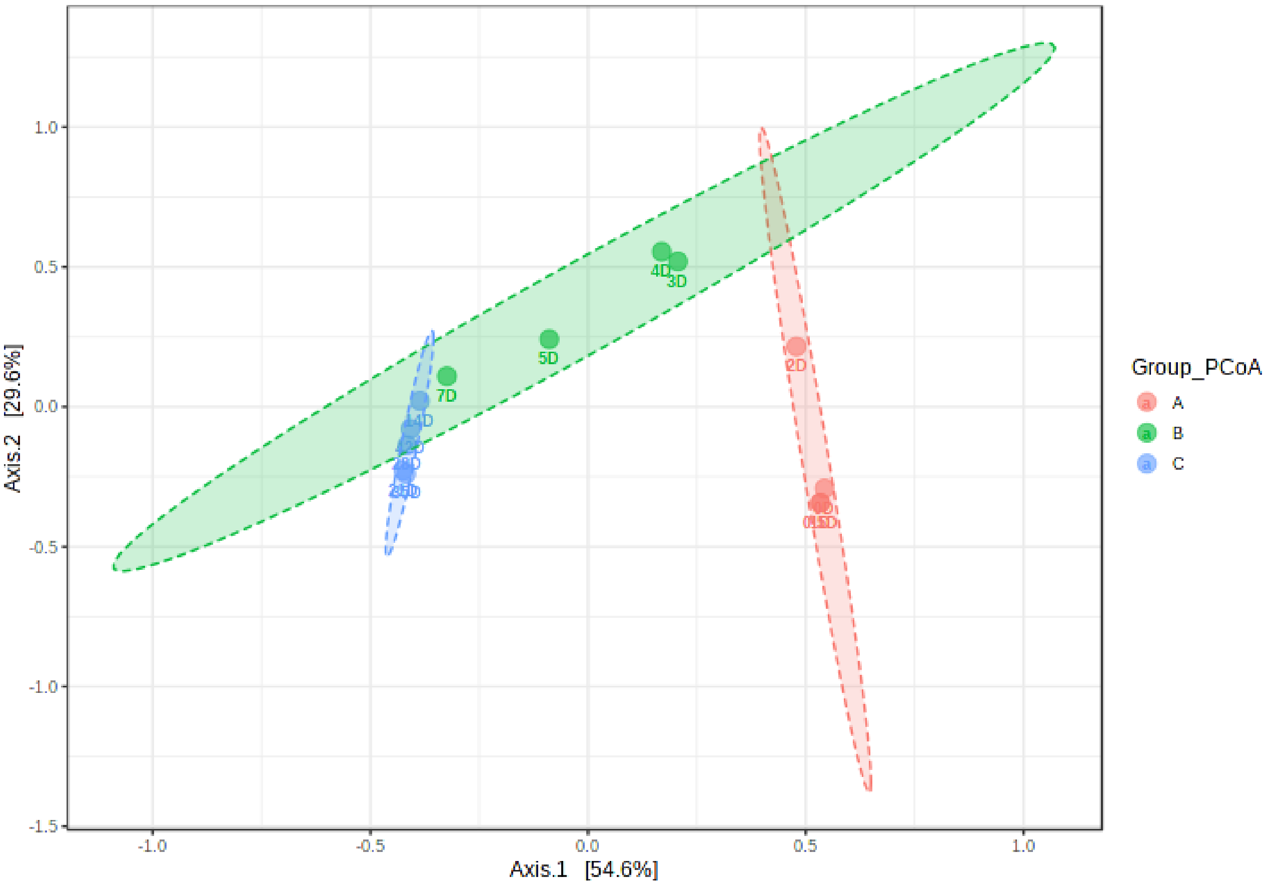

Axis.2 [29.6%]
Axis.1 [54.6%]
1.0
0.5
0.0
-0.5
-1.0
-1.5
-1.0
-0.5
0.0
0.5
1.0
4D
3D
5D
7D
2D
14D
Group_PCoA
A
B
C

Figure S2. Profile of microbial diversity at species level during spontaneous fermentation of sauerkraut carried out at 15 ºC for 42 days. Samples were taken at the beginning of fermentation (D0), after 0.5 (D0.5), 1 (D1), 2 (D2), 3 (D3), 4 (D4), 5 (D5), 7 (D7), 14 (D14), 21 (D21), 28 (D28), 35 (D35), and 42 (D42) days of fermentation. The colour shows the presence (Blue) or absence (Red) of species.

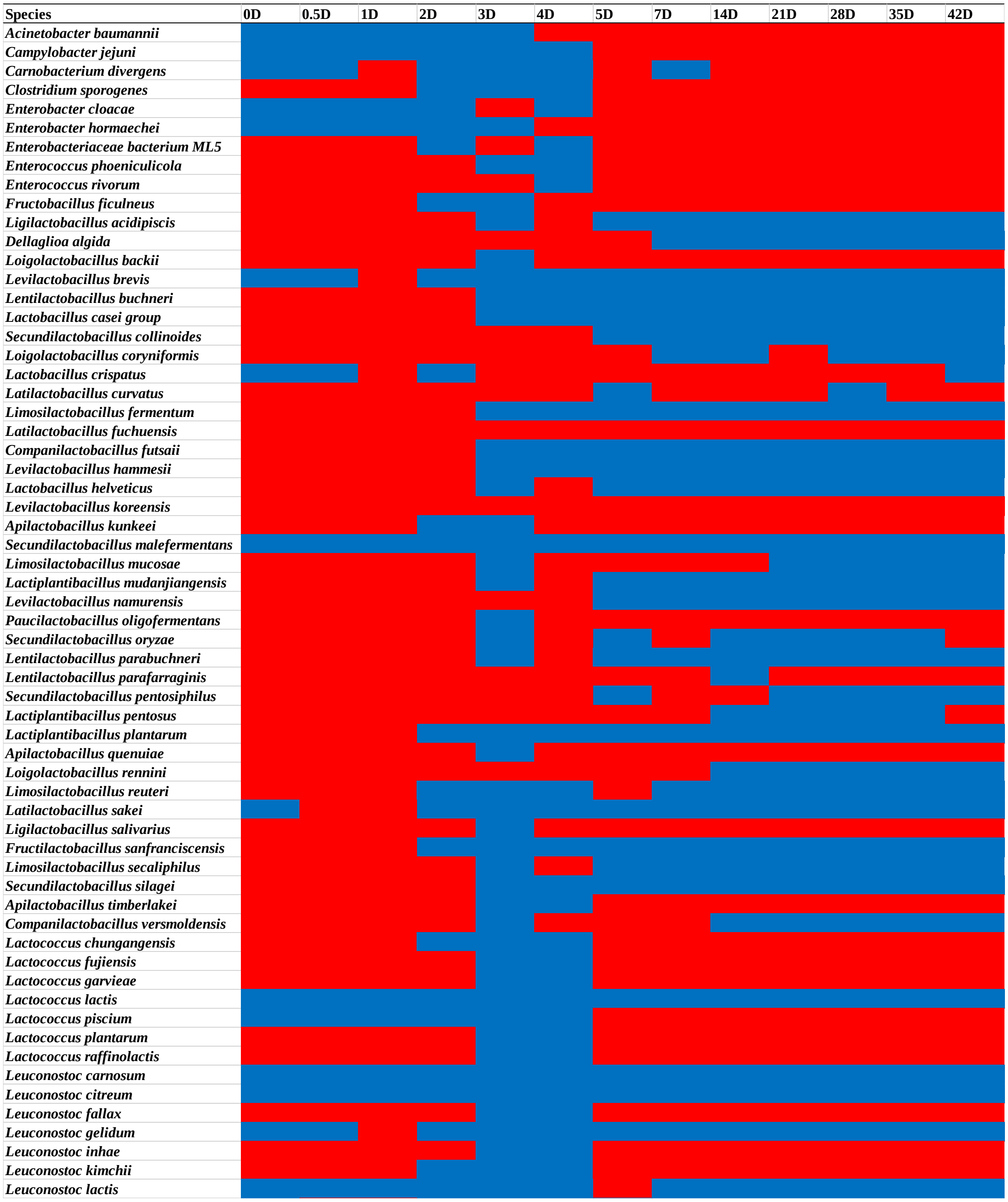

Figure S2. Continued.

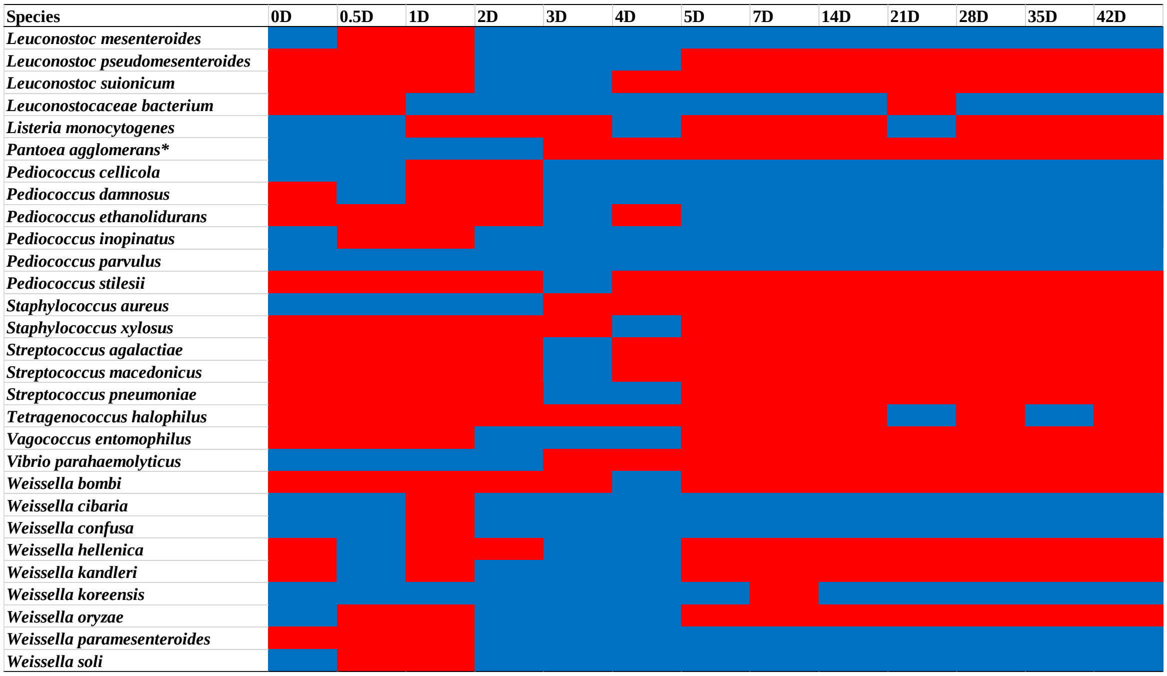

Figure S3. Lactic acid bacteria species profile of *vprA* (A) and *padR* (B) genes annotated correlated with phenolic compounds during spontaneous fermentation of sauerkraut at 15 ºC at different time points. The colour shows the numbers of coverage genes per species.

A

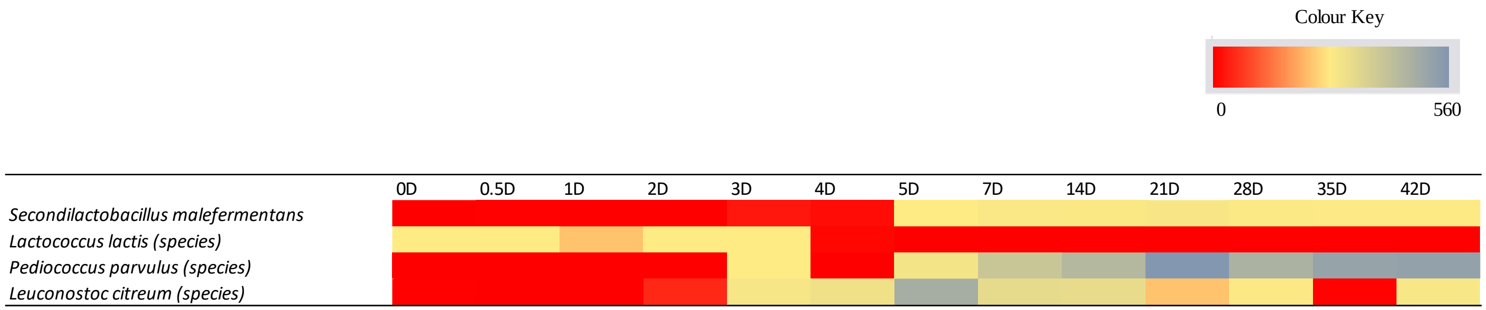

B

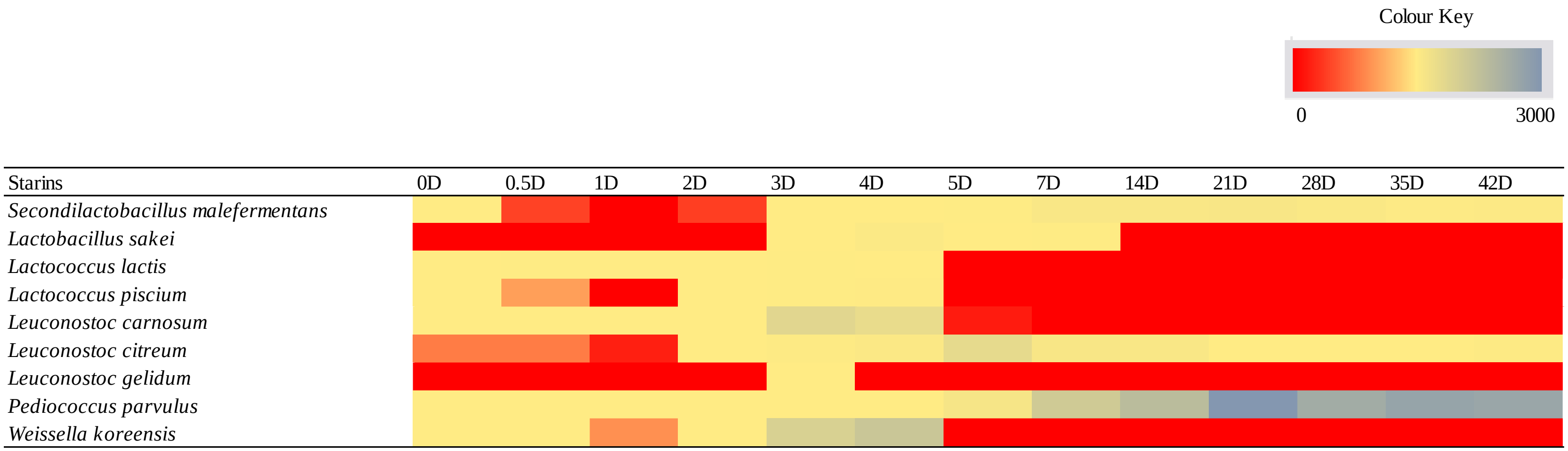
Colour Key
0
3000
Starins
0D
0.5D
1D
2D
3D
4D
5D
7D
14D
21D
28D
35D
42D
Secondilactobacillus malefermentans
Lactobacillus sakei
Lactococcus lactis
Lactococcus piscium
Leuconostoc carnosum
Leuconostoc citreum
Leuconostoc gelidum
Pediococcus parvulus
Weissella koreensis
